# HIV incidence, viremia, and the national response in Eswatini: Two sequential population-based surveys

**DOI:** 10.1371/journal.pone.0260892

**Published:** 2021-12-02

**Authors:** Rejoice Nkambule, Neena M. Philip, Giles Reid, Zandile Mnisi, Harriet Nuwagaba-Biribonwoha, Tony T. Ao, Choice Ginindza, Yen T. Duong, Hetal Patel, Suzue Saito, Chelsea Solmo, Kristin Brown, Chiara S. Moore, Andrew C. Voetsch, George Bicego, Naomi Bock, Fortune Mhlanga, Tengetile Dlamini, Khanya Mabuza, Amos Zwane, Ruben Sahabo, Trudy Dobbs, Bharat S. Parekh, Wafaa El-Sadr, Caroline Ryan, Jessica Justman

**Affiliations:** 1 Ministry of Health, Mbabane, Eswatini; 2 ICAP at Columbia University, Mailman School of Public Health, New York, NY, United States of America; 3 Department of Epidemiology, Columbia University, Mailman School of Public Health, New York, NY, United States of America; 4 ICAP at Columbia University, Mailman School of Public Health, Mbabane, Eswatini; 5 U.S. Centers for Disease Control and Prevention Eswatini, Mbabane, Eswatini; 6 Central Statistics Office, Mbabane, Eswatini; 7 U.S. Centers for Disease Control and Prevention, Center for Global Health, Division of Global HIV and TB, Atlanta, Georgia, United States of America; 8 PHI/CDC Global Health Fellowship Program, Oakland, CA, United States of America; 9 National Emergency Response Council on HIV/AIDS (NERCHA), Mbabane, Eswatini; Baylor College of Medicine, UNITED STATES

## Abstract

With the highest HIV incidence and prevalence globally, the government of Eswatini started a substantial scale-up of HIV treatment and prevention services in 2011. Two sequential large population-based surveys were conducted before and after service expansion to assess the impact of the national response. Cross-sectional, household-based, nationally representative samples of adults, ages 18 to 49 years, were sampled in 2011 and 2016. We measured HIV prevalence, incidence (recent infection based on limiting antigen ≤1.5 optical density units and HIV RNA ≥1000 copies/mL), viral load suppression (HIV RNA <1000 copies/mL among all seropositive adults) and unsuppressed viremia (HIV RNA ≥1000 copies/mL among all, regardless of HIV status) and assessed for temporal changes by conducting a trend analysis of the log ratio of proportions, using a Z statistic distribution. HIV prevalence remained stable from 2011 to 2016 [32% versus 30%, p = 0.10]. HIV incidence significantly declined 48% [2.48% versus 1.30%, p = 0.01]. Incidence remained higher among women than men [2011: 3.16% versus 1.83%; 2016: 1.76% versus 0.86%], with a smaller but significant relative reduction among women [44%; p = 0.04] than men [53%; p = 0.09]. The proportion of seropositive adults with viral load suppression significantly increased from 35% to 71% [p < .001]. The proportion of the total adult population with unsuppressed viremia decreased from 21% to 9% [p < .001]. National HIV incidence in Eswatini decreased by nearly half and viral load suppression doubled over a five-year period. Unsuppressed viremia in the total population decreased 58%. These population-based findings demonstrate the national impact of expanded HIV services in a hyperendemic country.

## Introduction

HIV remains a formidable global health threat. Thirty-eight million people were living with HIV (PLHIV) in 2019, and 1.7 million became newly infected. Nonetheless, substantial investment to stem the epidemic is yielding results. Global uptake of antiretroviral therapy (ART) more than tripled from 7.7 million in 2010 to 25.4 million in 2019, with a coinciding 39% reduction in HIV-related mortality [1]. These gains, coupled with the effectiveness of ART and other prevention methods to decrease new infections, have generated great optimism and motivated ambitious global targets to end the AIDS epidemic [2–6].

The Kingdom of Eswatini (formerly Swaziland), an African country of nearly 1.1 million people, has experienced the highest national levels of HIV [1, 7]. In 2011, the Swaziland HIV Incidence Measurement Survey (SHIMS) measured HIV prevalence at 32.1% [95% confidence interval (CI) (31.1, 33.0)] and prospectively observed HIV seroincidence at 2.38% [95% CI (2.1, 2∙8)] among adults ages 18 to 49 years [8, 9]. A parallel field validation of the limiting antigen-avidity (LAg-avidity) assay estimated HIV incidence at 2.5% [10]. Since then, Eswatini rapidly expanded its HIV programs with evidence-based interventions, including voluntary medical male circumcision (VMMC), targeted HIV testing campaigns, and decentralized, nurse-led ART initiation and monitoring. In light of the compelling findings on treatment as prevention and the global targets for ending the epidemic [6], expanded ART access became the cornerstone of Eswatini’s HIV response strategy [2, 11, 12]. By 2016, annual HIV testing had nearly tripled, and the cumulative number of PLHIV on treatment had more than doubled [13]. SHIMS was repeated in 2016, providing a unique opportunity to assess the national impact of five years of HIV program expansion through two population-based national surveys. We report here on the comparison of national estimates of HIV prevalence, incidence, and viral suppression among adults, ages 18 to 49 years, before and after service scale-up.

## Methods

### Study design

The two surveys consisted of unique cross-sectional, household-based, nationally representative samples assessed five years apart. Each sample was based on a two-stage, cluster-based design and varied in size due to different primary objectives and statistical power considerations. The Swaziland HIV Incidence Measurement Survey (S1 File) [8], was implemented between December 2010 and June 2011 among 14,891 selected households from 575 enumeration areas. The primary objective was to estimate HIV incidence among adults ages 18 to 49 years. SHIMS2 was implemented between October 2016 and March 2017 among 6,417 selected households from 286 enumeration areas. The primary objective was to estimate HIV incidence among adults ages 15 years and older. For this analysis, data were restricted to participants who reported being 18 to 49 years of age.

Selected households were approached by study staff trained in survey procedures and Good Clinical Practice [14]. Individuals who resided or had slept in the household the prior night and who were willing to answer questions in English or siSwati, receive home-based HIV testing, and provide written informed consent were eligible for participation. Both surveys were approved by ethical review boards at the Eswatini Ministry of Health, Columbia University Irving Medical Center, and the US Centers for Disease Control and Prevention.

### Procedures and outcomes

Survey staff collected information in or near participants’ homes on demographics, sexual behaviour, prior HIV diagnosis, ART initiation, and male circumcision status followed by HIV counselling, condom provision, and venipuncture. Household-based HIV rapid testing was done with immediate return of results and referral to HIV care for those testing HIV-seropositive.

Estimates of prior HIV diagnosis and current ART use were based on self-report. HIV prevalence estimates were calculated as a proportion using the number of individuals testing HIV-seropositive among all participants testing for HIV. Rapid HIV testing was conducted with Determine™ HIV-1/2 Ag/Ab Combo (in 2011) or Determine™ HIV-1/2 (in 2016). Determine-reactive samples were confirmed with Uni-Gold™ HIV Test. Laboratory-based confirmatory testing and resolution of discrepant results were conducted according to established test algorithms (S1 File). HIV incidence estimates were based on the number of HIV-seropositive samples categorized as recent, namely, the number of HIV-seropositive specimens with a LAg value ≤1.5 normalized optical density units and HIV RNA ≥1000 copies/mL [15–17]. As qualitative testing for detectable antiretrovirals was conducted only on 2016 samples, the incidence algorithm did not include absence of antiretrovirals. HIV viral load suppression estimates were calculated as a proportion using the number of HIV-seropositive individuals with a HIV viral load <1000 copies/mL among all participants testing HIV-seropositive. Unsuppressed HIV viremia estimates were calculated as a proportion using the number of HIV-seropositive individuals with a HIV viral load ≥1000 copies/mL among all participants, regardless of HIV serostatus.

### Statistical methods

The SHIMS1 sample size methodology is described elsewhere [8]. The SHIMS2 sample size was determined to obtain a national annual HIV incidence rate for adults, ages 15 to 49 years, targeting a relative standard error (RSE) of 20% or less. We used the CDC incidence calculator to identify a variance multiplier of 1.28, assuming HIV prevalence of 27.7%, annual HIV incidence of 1.89% [18], mean duration of recent infection of 130 days, proportion of false recents of 0% [17], and design effect of 1.48 based on an intra-cluster correlation of 0.05. We estimated 5,418 of the 6,417 selected households would yield an analytic sample of 8,167 adults ages 18 to 49 years, allowing 83% power to detect an estimated change from SHIMS1 in incidence of 43% or more.

Analysis methods were comparable between samples, incorporating design weights, based on selection probabilities of enumeration areas and households, as well as non-response and post-stratification adjustments. Standard errors and confidence intervals for population proportions were calculated using Taylor series estimation (S1 File) and jackknife replicate weights (S2 File). The probability of temporal differences in proportions assumed independence between surveys, using a Z statistic distribution with a delta approximation of the variance of the log ratio of the proportions. Missing values were excluded, and all proportions were calculated using final survey weights (S1 File).

## Results

### Survey response and participant characteristics

Of the 14,891 and 6,417 households selected for participation in 2011 (S1 File) and 2016 (S2 File), respectively, household response was 94% [12,571/13,335] in 2011 and 86% [5,185/6,056] in 2016 (S1 Table in S5 File). Weighted estimates show modest differences in the population profiles of adults, ages 18 to 49 years, in Eswatini between 2011 and 2016 (Table 1). The population in 2016 compared to 2011 was slightly younger [p < .001], more educated [p < .001], and more likely to be unemployed [p < .001]. Men were significantly more likely to be medically circumcised [17% versus 29%, risk ratio (RR) 1.69, 95% confidence interval (CI) (1.53, 1.86); p < .001]. While noting the different recall periods in the 2011 (six months) and 2016 (twelve months) surveys, reports of always using a condom increased from 2011 to 2016 [32% versus 40%]. Frequency of reporting two or more partners was similar [11% versus 12%].

**Table 1:** Population of adults, ages 18–49 years in Eswatini, 2011 and 2016*.

	2011			2016		
	Women	Men	Total	Women	Men	Total
	N (%)	N (%)	N (%)	N (%)	N (%)	N (%)
**Age (years)** [women (p < .001), men (p < .001), total (p < .001)]†						
18–19	1,199 (11)	1,005 (12)	2,204 (11)	420 (9)	418 (10)	838 (9)
20–24	2,808 (26)	1,950 (26)	4,758 (26)	950 (21)	709 (25)	1,659 (23)
25–29	2,217 (21)	1,417 (21)	3,634 (21)	861 (19)	642 (21)	1,503 (20)
30–34	1,509 (14)	1,043 (15)	2,552 (15)	811 (17)	541 (16)	1,352 (16)
35–39	1,191 (11)	674 (11)	1,865 (11)	602 (14)	453 (12)	1,055 (13)
40–44	1,061 (9)	543 (8)	1,604 (9)	450 (11)	323 (9)	773 (10)
45–49	975 (8)	449 (6)	1,424 (8)	420 (9)	246 (7)	666 (8)
**Residence** [women (p = .002), men (p < .001) and total (p < .001)]†						
Urban	3,172 (33)	2,051 (33)	5,223 (33)	1,100 (30)	867 (30)	1,967 (30)
Rural	7,788 (67)	5,030 (67)	12,818 (67)	3,414 (70)	2,465 (70)	5,879 (70)
**Education** [women (p < .001), men (p < .001), total (p < .001)]†						
Did not attend	722 (6)	454 (6)	1,176 (6)	142 (3)	104 (3)	246 (3)
Primary	3,230 (30)	1,964 (28)	5,194 (29)	1,062 (22)	822 (24)	1,884 (23)
Secondary	5,589 (51)	3,523 (49)	9,112 (50)	2,797 (62)	2,019 (61)	4,816 (62)
Tertiary	1,373 (13)	1,108 (16)	2,481 (14)	505 (12)	385 (13)	890 (12)
**Marital status** [women (p < .001), men (p < .001), total (p < .001)]†						
Married or living together	5,565 (51)	2,367 (36)	7,932 (45)	2,010 (44)	1,100 (33)	3,110 (39)
Not married, ever had sex	4,548 (43)	3,199 (47)	7,747 (44)	1,761 (40)	1,524 (48)	3,285 (44)
Not married, never had sex	668 (6)	1,285 (17)	1,953 (11)	330 (7)	491 (14)	821 (10)
Divorced/separated/widowed	-	-	-	376 (9)	155 (4)	531 (7)
**Employment status** [women (p < .001), men (p = .02), and total (p < .001)]†						
Employed	3,578 (37)	3,449 (60)	7,027 (47)	1,513 (35)	1,603 (48)	3,116 (41)
Unemployed	6,478 (63)	2,447 (40)	8,925 (53)	2,998 (65)	1,724 (52)	4,722 (59)
**Medical circumcision** [men (p < .001)]†						
Circumcised	-	1,174 (17)	-	-	930 (29)	-
Uncircumcised	-	5,622 (83)	-	-	2,364 (71)	-
**Frequency of condom use** ^¶^						
Always	2,563 (30)	1,625 (34)	4,188 (32)	812 (36)	732 (45)	1,544 (40)
Sometimes	2,966 (34)	1,866 (39)	4,832 (36)	1,059 (49)	714 (46)	1,773 (48)
Never	3,139 (36)	1,280 (27)	4,419 (32)	333 (15)	152 (9)	485 (12)
**Number of sexual partners** ^¶^						
0	1,422 (14)	975 (16)	2,397 (15)	770 (17)	710 (21)	1,480 (19)
1	8,480 (82)	3,640 (63)	12,120 (74)	3,410 (76)	1,917 (60)	5,327 (69)
2 or more	402 (4)	1,279 (21)	1,681 (11)	267 (6)	605 (19)	872 (12)

* Estimates are weighted population percentages and unweighted counts from Swaziland HIV Incidence Measurement Surveys (SHIMS) in 2011 and 2016

†P-value to test for difference in distribution between 2011 and 2016.

¶ The recall period for reported sexual behavior was 6 months in 2011 and 12 months in 2016. Therefore, statistical testing for distributional differences was not conducted.

### HIV epidemic measures

Between 2011 and 2016, national HIV prevalence remained stable [32% versus 30%, RR 0.95, 95% CI (0.90, 1.01); p = 0.10] and remained higher among women than men [39% versus 23% in 2011, 38% versus 21% in 2016; Table 2]. National HIV incidence significantly declined by 48% from 2011 to 2016 [2.48% versus 1.30%, RR 0.52, 95% CI (0.33, 0.84); p = 0.01], with a significant reduction among women (44%, p = 0.04) but not among men (53%, p = 0.09). HIV incidence was significantly higher among women than men in 2011 [3.16% versus 1.83%, RR 1.73, 95% CI (1.14–2.62); p = .01] and remained comparatively greater in 2016 [1.76% versus 0.86%] but with a non-significant difference [RR 2.05, 95% CI (0.79–5.28); p = .14]. HIV incidence significantly decreased among 18- to 24-year-old women [40%, p = .03] (S2 Table in S5 File).

**Table 2:** HIV prevalence, incidence, viral load suppression, and unsuppressed viremia among adults 18–49 years in Eswatini, 2011 and 2016.

	2011		2016		2011 versus 2016	
	N	% (95% CI)	N	% (95% CI)	Risk ratio (95% CI)	p-value
**HIV prevalence**						
**Total**	18,172	32 (31, 33)	7,265	30 (29, 32)	0.95 (0.90, 1.01)	0.10
**Women**	11,042	39 (38, 40)	4,252	38 (36, 40)	0.98 (0.91, 1.06)	0.60
**Men**	7,130	23 (22, 25)	3,013	21 (19, 23)	0.88 (0.77, 1.01)	0.10
HIV incidence*						
**Total**	17,975	2.48 (1.95, 3.01)	7,264	1.30 (0.75, 1.85)	0.52 (0.33, 0.84)	0.01
**Women**	10,914	3.16 (2.39, 3.92)	4,251	1.76 (0.87, 2.65)	0.56 (0.32, 0.98)	0.04
**Men**	7,061	1.83 (1.21, 2.45)	3,013	0.86 (0.16, 1.55)	0.47 (0.20, 1.12)	0.09
**HIV viral load suppression prevalence among all HIV-seropositive adults** ^†^						
**Total**	5,686	35 (33, 36)	2,361	71 (69, 73)	2.05 (1.95, 2.15)	< 0.001
**Women**	4,146	36 (34, 37)	1,659	75 (72, 78)	2.09 (1.91, 2.28)	< 0.001
**Men**	1,540	33 (30, 35)	702	63 (59, 67)	1.93 (1.62, 2.29)	< 0.001
**Unsuppressed HIV viremia prevalence among all, regardless of HIV status** ^‡^						
**Total**	17,997	21 (20, 21)	7,265	9 (8, 10)	0.42 (0.38, 0.47)	< 0.001
**Women**	10,929	25 (24, 26)	4,252	10 (8, 11)	0.39 (0.34, 0.44)	< 0.001
**Men**	7,068	16 (15, 17)	3,013	8 (7, 9)	0.49 (0.42, 0.57)	< 0.001

* Measured using LAg avidity assay and HIV viral load

† Defined as proportion of persons with HIV viral load <1000 copies/mL in the population testing HIV seropositive

‡ Defined as proportion of persons with HIV viral load ≥1000 copies/mL in the total population, regardless of HIV status

The prevalence of viral load suppression among all PLHIV more than doubled from 35% in 2011 to 71% in 2016 [RR 2.05, 95% CI (1.95, 2.15); p < .001], with significant increases in women [36% versus 75%; RR 2.09, 95% CI (1.91, 2.28); p < .001] and men [33% versus 63%; RR 1.93, 95% CI (1.62, 2.29); p < .001]. The proportion with unsuppressed viremia among the national population, regardless of HIV serostatus, significantly decreased by 57% between 2011 and 2016 [21% versus 9%; RR 0.42, 95% CI (0.38, 0.47); p < .001].

### HIV care continuum measures

**Fig 1:**
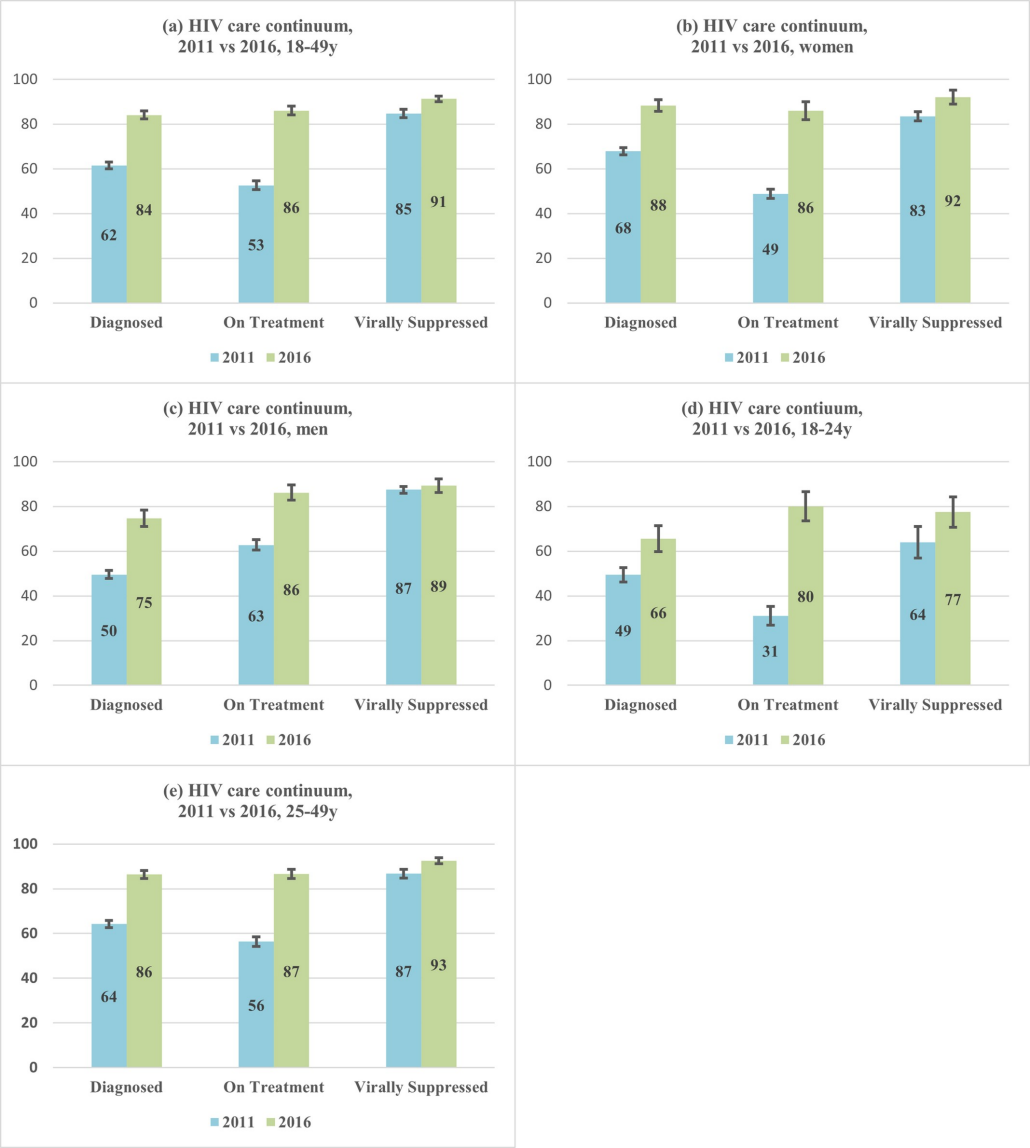
HIV care continuum (conditional proportions)* by age and by sex in 2011 and 2016. * Diagnosed was defined as self-reporting HIV-positive. Among those diagnosed, on treatment was defined as self-reporting current use of ART. Among those on treatment, virally suppressed was defined as viral load <1000 copies/mL.

Among those testing HIV-seropositive in each survey, prevalence of prior HIV diagnosis significantly grew from 62% in 2011 to 84% in 2016 [RR 1.37, 95% CI (1.32, 1.41); p < .001], with a significant increase among women [20%, p < .001] and larger significant increase among men [25%, p < .001] (Fig 1 and S3 Table in S5 File). Prevalence of prior HIV diagnosis significantly increased among 18- to 24-year-olds [49% versus 66%, p < .001] and among 25- to-49-year olds [64% versus 86%, p < .001].

Among those reporting a prior diagnosis, the conditional prevalence of reporting current ART use significantly increased from 53% in 2011 to 86% in 2016 [RR 1.64, 95% CI (1.56, 1.71); p < .001]. This was observed among women [49% versus 86%; p < .001] and men [63% versus 86%; p < .001] with a larger significant increase among younger individuals [18- to-24-year olds: 31% in 2011 to 80% in 2016, p < .001] versus older individuals [25- to-49-year olds: 56% in 2011 to 87% in 2016, p < .001].

Among those reporting current ART use, the conditional prevalence of viral load suppression significantly grew from 85% in 2011 to 91% in 2016 [RR 1.08, 95% CI (1.05,1.10); p < .001]. The increase in viral suppression prevalence was significant among women [83% versus 92%; RR 1.10, 95% CI (1.07, 1.14); p < .001] but not among men [87% versus 89%; RR 1.02, 95% CI (0.97, 1.07); p = 0.40)]. While viral load suppression was more common among older compared to younger persons in 2011 and 2016, a significant increase was observed from 2011 to 2016 among 18- to 24-year-olds [64% versus 77%; RR 1.21, 95% CI (1.05, 1.39); p = .01] but not among 25- to 49-year-olds [87% versus 93%; RR 1.07, 95% CI (0.98, 1.16); p = .10].

We also assessed the changes in unconditional measures of the HIV care continuum among all seropositive persons in 2011 and 2016 by sex-specific age groups (Table 3). While women and men in both age groups achieved significant increases in all comparisons, the largest relative increases between surveys occurred among young people. From 2011 to 2016, prevalence of viral load suppression among all seropositive 18- to 24-year-olds was four and sevenfold greater among women [RR 4.06, CI (3.18, 5.17); p < .001] and men [RR 7.02, CI (2.59, 19.02); p < .001], respectively, versus a twofold increase among all seropositive 25- to 49-year-old women [RR 2.24, CI (2.10, 2.40); p < .001] and men [RR 2.06, CI (1.84, 2.30); p < .001]. Nevertheless, viral load suppression in 18- to 24-year-olds remains substantially lower than in 25- to 49-year-olds [women: 44% versus 74%; men: 27% versus 60%, respectively].

**Table 3:** Univariate prevalence ratios and 95% CIs for association between age group and HIV continuum of care measures among all persons testing HIV-positive by sex in 2011 and 2016.

	Women				Men			
	2011 N (%)	2016 N (%)	Risk Ratio (CI)	P-value	2011 N (%)	2016 N (%)	Risk Ratio (CI)	P-value
	**Prior diagnosis of HIV**							
18–24 years	1,037 (54)	233 (70)	1.30 (1.17–1.45)	< .001	131 (22)	42 (47)	2.10 (1.30–3.41)	0.003
25–49 years	3,129 (72)	1,419 (91)	1.26 (1.23–1.29)	< .001	1,419 (52)	658 (77)	1.48 (1.38–1.59)	< .001
	**Currently on treatment**							
18–24 years	1,037 (17)	233 (54)	3.29 (2.70–4.00)	< .001	131 (8)	42 (44)	5.73 (2.82–11.67)	< .001
25–49 years	3,129 (38)	1,419 (79)	2.07 (1.95–2.19)	< .001	1,419 (33)	658 (66)	2.00 (1.81–2.21)	< .001
	**Virally suppressed**							
18–24 years	1,037 (11)	233 (44)	4.06 (3.18–5.17)	< .001	131 (4)	42 (27)	7.02 (2.59–19.02)	< .001
25–49 years	3,129 (33)	1,419 (74)	2.24 (2.10–2.40)	< .001	1,419 (29)	658 (60)	2.06 (1.84–2.30)	< .001

## Discussion

We describe one of the first direct measures of the population-level impact of HIV program expansion on national HIV incidence, population viremia, and viral suppression. Two rigorous population-based surveys conducted five years apart in Eswatini, the country with the highest levels of HIV in the world, show significant growth in national ART coverage paralleled by a twofold increase in the proportion of PLHIV with viral load suppression and a decrease in HIV incidence by nearly half, providing the first quantified evidence for the effectiveness of treatment scale-up on HIV incidence at a national level.

This decline in HIV incidence most likely resulted from the rapid expansion of ART services, an explanation supported by the observed doubling of national viral load suppression from 35% to 71%. Expanded treatment increased viral suppression, leading to fewer incident cases. The almost 60% decrease in the prevalence of unsuppressed HIV viremia among the entire population (regardless of HIV status) signifies a substantial decline in HIV transmission risk in the population [19]. Despite this remarkable progress, national HIV incidence in 2016, 1.30%, remained very high, underscoring the continued need for enhanced expansion of HIV treatment programs and scale-up of primary prevention services.

**Fig 2:**
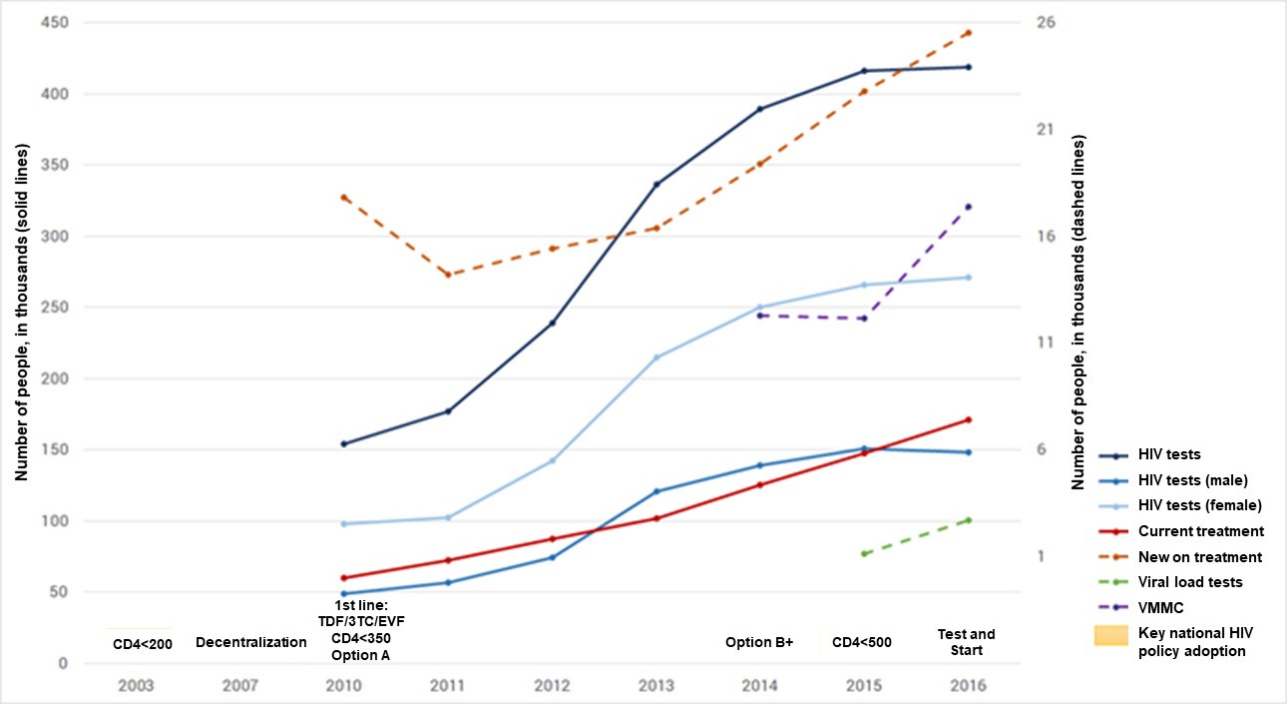
Key national HIV policy and program achievements, Eswatini, 2003–2016 [13]. Black solid line: HIV tests; blue solid line: HIV tests (Male); light blue solid line: HIV tests (Female); red solid line: currently on treatment; orange dotted line: new on treatment; green dotted line: viral load tests; purple dotted line: VMMC; text boxes: key national HIV policy adoption.

Several national policies were instituted in Eswatini between 2011 and 2016. Decentralization of HIV programs was enhanced, broadening access in rural areas. HIV testing points increased through task-shifting to lay test counsellors and growth in community-based testing. These boosted case-finding efforts resulted in a near tripling in the number of HIV tests conducted from 2011 to 2016 (Fig 2). ART access was increased through task-shifting of ART services from physicians to nurses and the expansion of treatment eligibility to all persons diagnosed with HIV, based on WHO guideline updates [20]. These efforts aimed to maximize the potential of ART and viral suppression on HIV transmission, as noted among clinical trials of serodiscordant couples and in mathematical modelling [2, 21–23]. Cohort studies in concentrated and generalized epidemics have shown the impact of ART on number of new infections and HIV incidence [24–32].

More recently, community randomized trials of “universal test and treat” have shown inconsistent results regarding the impact on HIV incidence at a community level, potentially due to limits in ART coverage in hard-to-reach populations, like men and young people, both within and outside of evaluated communities [33–37]. Our findings, however, show men demonstrating a larger relative increase than women in prior HIV diagnosis from 2011 to 2016, leading to significant increases in ART coverage and viral load suppression among all HIV-positive men. The increased engagement of men in Eswatini may have prompted the significant decline in new infections among women, which drove the overall reduction in national HIV incidence. Combined with the significant growth in ART uptake and viral suppression among 25- to 49-year-old women, which likely curbed the number of new infections among 25- to 34-year old men who had the highest risk of incident infection in 2011, the sex differential in incidence in 2011 became nonsignificant (potentially limited by sample size) by 2016 [9, 38].

Another key finding from our study is the notable improvement in ART use among young people. From 2011 to 2016, 18- to 24-year-olds exhibited a greater relative increase of current ART use among those previously diagnosed than compared with 25- to 49-year-olds, and the proportion of all HIV-seropositive 18- to 24-year-old women and men on ART grew over three and fivefold, respectively. The proportion of all HIV-seropositive 18- to 24-year-old women and men with viral suppression in 2011 multiplied by four and seven times, respectively, by 2016. Taken together, these findings provide encouraging news that young people and men can be engaged in sustained HIV care, setting a future pathway for reduced population-level HIV transmission risk and continued reductions in incidence in hyperendemic countries on track to achieving global targets [37, 38].

Other factors seem less plausible to substantially account for the observed sizable decrease in HIV incidence. VMMC coverage among men likely had limited effect on HIV incidence across the general population, since impact manifests over a minimum 10-year period in populations with comparatively higher VMMC prevalence, relatively higher levels of risk behaviors, and comparatively lower HIV prevalence than Eswatini [39, 40]. The changes in sexual risk behaviors may have modestly contributed to the change in HIV incidence; but interpretation is limited by measurement differences between surveys, and caution is justified when interpreting self-reported sexual behaviors. Lastly, underlying shifts in national demographics seem unlikely since comparison of demographic profiles of the national population, while statistically different, showed minimal meaningful differences in age and marital distributions between surveys.

Despite the impressive progress in hard-to-reach populations, substantial gaps remain among men, particularly young men. In 2016, prior HIV diagnosis among HIV-positive men still lagged behind women (75% versus 88%); more than half of HIV-positive young men (53%) were unaware of their HIV status, and only 27% of those aware and on ART had viral suppression, compared to 44% of young women. These findings provide important context for the continued high national incidence in Eswatini, highlighting the need for male- and youth-friendly HIV services for this sizable segment of the population.

This study has several strengths. Results are derived from large, population-based, nationally-representative samples distinguished by rigorous biomarker testing and high participation rates, providing robust outcome and external validity to our results. However, study findings may have been affected by self-report biases regarding prior HIV diagnosis and ART uptake due to stigma, which may have under-reported knowledge of HIV status and either under or over-reported ART coverage. Also, the lack of antiretroviral detection in the incidence algorithm, when combined with an assumed false recency rate of 0%, may have resulted in a small proportion of long-term infections misclassified as recent in both surveys. However, use of a non-zero FRR would be inappropriate, given the differences in FRR among populations over time and that a non-zero FRR reduces recent cases without improving the positive predictive value of the algorithm [41]. Lastly, population-level causality between population-level viral load suppression and HIV incidence is difficult to establish without identification and measurement of population-level mechanisms; for example, unmeasured factors, such as transmission risk related to population mobility within sexual networks, may have affected the levels of new HIV infections.

In conclusion, expansion of HIV services in Eswatini between 2011 to 2016 coincided with a reduction in HIV transmission in the country by nearly half. This is the first national-level demonstration that HIV treatment, when scaled countrywide, is associated with substantial reductions in unsuppressed HIV viremia and new infections. These findings show the feasibility in low-resource, hyperendemic countries to rapidly expand HIV services and dramatically alter epidemics. Further efforts are needed in Eswatini to extend these achievements towards universal ART coverage for all PLHIV and to continue expansion of services focused on the subpopulations that have yet to benefit from these interventions.

## Supporting information

S1 File SHIMS1 2011 questionnaire. (PDF)

S2 File SHIMS2 2016 questionnaire. (PDF)

S3 File SHIMS1-2 anonymized, minimal dataset. (CSV)

S4 File SHIMS1-2 dataset codebook. (XLSX)

S5 File (DOCX)

## References

[1] UNAIDS. Global HIV & AIDS statistics—2020 fact sheet. Geneva: UNAIDS; 2021 [cited 2021 March 4]. Available from: https://www.unaids.org/sites/default/files/media_asset/UNAIDS_FactSheet_en.pdf.

[2] Cohen MS, Chen YQ, McCauley M, Gamble T, Hosseinipour MC, Kumarasamy N, et al. Prevention of HIV-1 infection with early antiretroviral therapy. New England Journal of Medicine. 2011;365(6):493–505. doi: 10.1056/NEJMoa1105243 ; PubMed Central PMCID: PMC3200068. 21767103 PMC3200068

[3] Auvert B, Taljaard D, Lagarde E, Sobngwi-Tambekou J, Sitta R, Puren A. Randomized, controlled intervention trial of male circumcision for reduction of HIV infection risk: the ANRS 1265 Trial. PLoS Medicine. 2005;2(11):e298. doi: 10.1371/journal.pmed.0020298 ; PubMed Central PMCID: PMC1262556. 16231970 PMC1262556

[4] Bailey RC, Moses S, Parker CB, Agot K, Maclean I, Krieger JN, et al. Male circumcision for HIV prevention in young men in Kisumu, Kenya: a randomised controlled trial. Lancet. 2007;369(9562):643–56. doi: 10.1016/S0140-6736(07)60312-2 . 17321310

[5] Gray RH, Kigozi G, Serwadda D, Makumbi F, Watya S, Nalugoda F, et al. Male circumcision for HIV prevention in men in Rakai, Uganda: a randomised trial. Lancet. 2007;369(9562):657–66. doi: 10.1016/S0140-6736(07)60313-4 . 17321311

[6] UNAIDS. 90-90-90 an ambitious treatment target to help end the AIDS epidemic. Geneva, Switzerland: Joint United Nations Programme on HIV/AIDS, 2014.

[7] 2017 Population and Housing Census Preliminary Report. Mbabane, Swaziland: Central Statistics Office, 2017.

[8] Bicego GT, Nkambule R, Peterson I, Reed J, Donnell D, Ginindza H, et al. Recent patterns in population-based HIV prevalence in Swaziland. PloS One. 2013;8(10):e77101. doi: 10.1371/journal.pone.0077101 ; PubMed Central PMCID: PMC3797108. 24143205 PMC3797108

[9] Justman J, Reed JB, Bicego G, Donnell D, Li K, Bock N, et al. Swaziland HIV Incidence Measurement Survey (SHIMS): a prospective national cohort study. Lancet HIV. 2017;4(2):e83–e92. doi: 10.1016/S2352-3018(16)30190-4 ; PubMed Central PMCID: PMC5291824. 27863998 PMC5291824

[10] Pottinger YD DT, Mavengere Y, Manjengwa J, Rottinghaus E, Saito S, Bock N, et al. Field validation of limiting-antigen avidity enzyme immunoassay to estimate HIV-1 incidence in cross-sectional survey in Swaziland. AIDS Research and Human Retroviruses. 2019. doi: 10.1089/AID.2018.0284 31204867 PMC11301577

[11] Lundgren JD, Babiker AG, Gordin F, Emery S, Sharma S, Avihingsanon A-C, et al. Initiation of Antiretroviral Therapy in Early Asymptomatic HIV Infection. New England Journal of Medicine. 2015;373(9):795–807. doi: 10.1056/NEJMoa1506816 PubMed PMID: WOS:000360171700006. 26192873 PMC4569751

[12] Danel C, Moh R, Gabillard D, Badje A, Le Carrou J, Ouassa T, et al. A Trial of Early Antiretrovirals and Isoniazid Preventive Therapy in Africa. New England Journal of Medicine. 2015;373(9):808–22. Epub 2015/07/21. doi: 10.1056/NEJMoa1507198 . 26193126

[13] PEPFAR monitoring, Evaluation, and Reporting (MER) Data. Eswatini, 2003–2016. Washington D.C.: President’s Emergency Plan for AIDS Relief; 2019. Available from: https://data.pepfar.gov.

[14] Dixon JR. The International Conference on Harmonization Good Clinical Practice Guideline. Quality Assurance. 1999;6(2):65–74. doi: 10.1080/105294199277860 10386329

[15] Technical update on HIV incidence assays for surveillance and monitoring purposes. Geneva, Switzerland: UNAIDS and WHO, 2015.

[16] Duong YT, Qiu M, De AK, Jackson K, Dobbs T, Kim AA, et al. Detection of recent HIV-1 infection using a new limiting-antigen avidity assay: potential for HIV-1 incidence estimates and avidity maturation studies. PloS One. 2012;7(3):e33328. Epub 2012/04/06. doi: 10.1371/journal.pone.0033328 ; PubMed Central PMCID: PMC3314002. 22479384 PMC3314002

[17] Duong YT, Kassanjee R, Welte A, Morgan M, De A, Dobbs T, et al. Recalibration of the limiting antigen avidity EIA to determine mean duration of recent infection in divergent HIV-1 subtypes. PloS One. 2015;10(2):e0114947. Epub 2015/02/25. doi: 10.1371/journal.pone.0114947 ; PubMed Central PMCID: PMC4339840. 25710171 PMC4339840

[18] UNAIDS. UNAIDS 2015 estimates 2015. Available from: http://www.unaids.org/en/resources/documents/2017/HIV_estimates_with_uncertainty_bounds_1990-2016).

[19] Vandormael A, Barnighausen T, Herbeck J, Tomita A, Phillips A, Pillay D, et al. Longitudinal Trends in the Prevalence of Detectable HIV Viremia: Population-Based Evidence From Rural KwaZulu-Natal, South Africa. Clinical Infectious Diseases. 2018;66(8):1254–60. doi: 10.1093/cid/cix976 ; PubMed Central PMCID: PMC5889002. 29186391 PMC5889002

[20] WHO. Consolidated guidelines on the use of antiretroviral drugs for treating and preventing HIV infection: recommendations for a public health approach. Geneva, Switzerland: World Health Organization; 2013. 24716260

[21] Granich RM, Gilks CF, Dye C, De Cock KM, Williams BG. Universal voluntary HIV testing with immediate antiretroviral therapy as a strategy for elimination of HIV transmission: a mathematical model. Lancet. 2009;373(9657):48–57. doi: 10.1016/S0140-6736(08)61697-9 . 19038438

[22] Dodd PJ, Garnett GP, Hallett TB. Examining the promise of HIV elimination by ’test and treat’ in hyperendemic settings. AIDS. 2010;24(5):729–35. doi: 10.1097/QAD.0b013e32833433fe ; PubMed Central PMCID: PMC2852517. 20154580 PMC2852517

[23] Hontelez JA, Lurie MN, Barnighausen T, Bakker R, Baltussen R, Tanser F, et al. Elimination of HIV in South Africa through expanded access to antiretroviral therapy: a model comparison study. PLoS Medicine. 2013;10(10):e1001534. doi: 10.1371/journal.pmed.1001534 ; PubMed Central PMCID: PMC3805487. 24167449 PMC3805487

[24] Montaner JS, Lima VD, Barrios R, Yip B, Wood E, Kerr T, et al. Association of highly active antiretroviral therapy coverage, population viral load, and yearly new HIV diagnoses in British Columbia, Canada: a population-based study. Lancet. 2010;376(9740):532–9. doi: 10.1016/S0140-6736(10)60936-1 ; PubMed Central PMCID: PMC2996043. 20638713 PMC2996043

[25] Das M, Chu PL, Santos GM, Scheer S, Vittinghoff E, McFarland W, et al. Decreases in community viral load are accompanied by reductions in new HIV infections in San Francisco. PloS One. 2010;5(6):e11068. doi: 10.1371/journal.pone.0011068 ; PubMed Central PMCID: PMC2883572. 20548786 PMC2883572

[26] Okano JT, Robbins D, Palk L, Gerstoft J, Obel N, Blower S. Testing the hypothesis that treatment can eliminate HIV: a nationwide, population-based study of the Danish HIV epidemic in men who have sex with men. Lancet Infectious Diseases. 2016;16(7):789–96. doi: 10.1016/S1473-3099(16)30022-6 ; PubMed Central PMCID: PMC5242518. 27174504 PMC5242518

[27] Tanser F, Baernighausen T, Grapsa E, Zaidi J, Newell M-L. High Coverage of ART Associated with Decline in Risk of HIV Acquisition in Rural KwaZulu-Natal, South Africa. Science. 2013;339(6122):966–71. doi: 10.1126/science.1228160 PubMed PMID: WOS:000315149600052. 23430656 PMC4255272

[28] Tanser F, Vandormael A, Cuadros D, Phillips AN, de Oliveira T, Tomita A, et al. Effect of population viral load on prospective HIV incidence in a hyperendemic rural African community. Science Translational Medicine. 2017;9(420). doi: 10.1126/scitranslmed.aam8012 . 29237762 PMC6435884

[29] Borgdorff MW, Kwaro D, Obor D, Otieno G, Kamire V, Odongo F, et al. HIV incidence in western Kenya during scale-up of antiretroviral therapy and voluntary medical male circumcision: a population-based cohort analysis. Lancet HIV. 2018;5(5):e241–e9. doi: 10.1016/S2352-3018(18)30025-0 . 29650451

[30] Oldenburg CE, Barnighausen T, Tanser F, Iwuji CC, De Gruttola V, Seage GR 3rd, et al. Antiretroviral Therapy to Prevent HIV Acquisition in Serodiscordant Couples in a Hyperendemic Community in Rural South Africa. Clinical Infectious Diseases. 2016;63(4):548–54. doi: 10.1093/cid/ciw335 ; PubMed Central PMCID: PMC4967606. 27208044 PMC4967606

[31] Grabowski MK, Serwadda DM, Gray RH, Nakigozi G, Kigozi G, Kagaayi J, et al. HIV Prevention Efforts and Incidence of HIV in Uganda. New England Journal of Medicine. 2017;377(22):2154–66. doi: 10.1056/NEJMoa1702150 ; PubMed Central PMCID: PMC5627523. 29171817 PMC5627523

[32] South African National HIV Prevalence, Incidence, Behavior and Communication Survey, 2017 Pretoria, South Africa: The Human Sciences Research Council; [cited 2019 August 4]. Available from: http://www.hsrc.ac.za/uploads/pageContent/9234/FINAL%20Presentation%20for%2017%20July%20launch.pdf.

[33] Iwuji CC, Orne-Gliemann J, Larmarange J, Balestre E, Thiebaut R, Tanser F, et al. Universal test and treat and the HIV epidemic in rural South Africa: a phase 4, open-label, community cluster randomised trial. Lancet HIV. 2018;5(3):e116–e25. doi: 10.1016/S2352-3018(17)30205-9 . 29199100

[34] Havlir DV, Balzer LB, Charlebois ED, Clark TD, Kwarisiima D, Ayieko J, et al. HIV Testing and Treatment with the Use of a Community Health Approach in Rural Africa. New England Journal of Medicine. 2019;381(3):219–29. Epub 2019/07/18. doi: 10.1056/NEJMoa1809866 . 31314966 PMC6748325

[35] Hayes RJ, Donnell D, Floyd S, Mandla N, Bwalya J, Sabapathy K, et al. Effect of Universal Testing and Treatment on HIV Incidence—HPTN 071 (PopART). New England Journal of Medicine. 2019;381(3):207–18. Epub 2019/07/18. doi: 10.1056/NEJMoa1814556 ; PubMed Central PMCID: PMC6587177. 31314965 PMC6587177

[36] Makhema J, Wirth KE, Pretorius Holme M, Gaolathe T, Mmalane M, Kadima E, et al. Universal Testing, Expanded Treatment, and Incidence of HIV Infection in Botswana. New England Journal of Medicine. 2019;381(3):230–42. Epub 2019/07/18. doi: 10.1056/NEJMoa1812281 . 31314967 PMC6800102

[37] Abdool Karim SS. HIV-1 Epidemic Control—Insights from Test-and-Treat Trials. New England Journal of Medicine. 2019;381(3):286–8. Epub 2019/07/18. doi: 10.1056/NEJMe1907279 . 31314975

[38] De Oliveira T, Kharsany AB, Graf T, Cawood C, Khanyile D, Grobler A, et al. Transmission networks and risk of HIV infection in KwaZulu-Natal, South Africa: a community-wide phylogenetic study. Lancet HIV. 2017;4(1):e41–e50. Epub 2016/12/05. doi: 10.1016/S2352-3018(16)30186-2 ; PubMed Central PMCID: PMC5479933. 27914874 PMC5479933

[39] Kong X, Kigozi G, Ssekasanvu J, Nalugoda F, Nakigozi G, Ndyanabo A, et al. Association of Medical Male Circumcision and Antiretroviral Therapy Scale-up With Community HIV Incidence in Rakai, Uganda. JAMA. 2016;316(2):182–90. doi: 10.1001/jama.2016.7292 ; PubMed Central PMCID: PMC5027874. 27404186 PMC5027874

[40] UNAIDS. Male circumcision for HIV prevention in high HIV prevalence settings: what can mathematical modelling contribute to informed decision making? PLoS Medicine. 2009;6(9):e1000109. doi: 10.1371/journal.pmed.1000109 ; PubMed Central PMCID: PMC2731851. 19901974 PMC2731851

[41] Voetsch AC, Duong YT, Stupp P, Saito S, McCracken S, Dobbs T et al. HIV-1 recent infection testing algorithm with antiretroviral drug detection to improve accuracy of incidence estimates. Journal of Acquired Immune Deficiency Syndrome. 2021;87(Suppl 1):S73–S80. doi: 10.1097/QAI.0000000000002707 . 34166315 PMC8630595

