# Supplementary material for: HIV incidence, viremia, and the national response in Eswatini: Two sequential population-based surveys
Source: PLoS One. 2021 Dec 2;16(12):e0260892. doi: 10.1371/journal.pone.0260892 (PMC8639055; doi:10.1371/journal.pone.0260892)
Supplement: S1 File — (PDF) [file pone.0260892.s001.pdf]

PTID ☐-☐☐☐-☐☐-☐-☐☐

Routine Questionnaire (A1)

Enrollment Date ☐☐ *dd* ☐☐☐ *MMM* ☐☐ *yy*

Staff ID: ☐☐☐ ☐☐ *Team ID:* ☐☐

Instructions: *Use this Routine Questionnaire for all participants meeting the eligibility criteria who have provided written informed consent to participate. Please do not leave any questions blank. Instead, mark the "DK" box if the participant states that they "don't know" the answer to a question. If the participant is willing to answer but doesn't know the exact answer, encourage him/her to estimate, as this is better than a DK answer. If the participant refuses to answer a question, mark the "REF" box for "refused" to answer.*

1. *Mark the sex of participant:* ☐ *male* ☐ *female*

Interviewer reads:

Thank you for agreeing to participate. First, I would like to ask you a few questions. Some of these questions may be uncomfortable to answer. Please remember that you do not have to answer any questions that you do not want to answer and you may discontinue the interview at any time. If I ask a question that you don't want to answer, just let me know and I will go on to the next question. Our discussion will last no more than 30 minutes.

2. In what month and year were you born? ☐☐☐ *MMM* ☐☐ *yy* → *If unknown, record age at last birthday: Estimate OK.* ☐☐ *years*

3. What is the highest level of school you attended? ☐ *primary* ☐ *secondary* ☐ *higher* ☐ *did not attend* ☐ *DK* ☐ *REF* → *If did not attend, DK, or REF, skip to 4.*

3a. What is the highest grade/form/year you completed at that level? ☐☐ *years* ☐ *DK* ☐ *REF*

PTID

☐-☐☐☐-☐☐-☐-☐☐

Routine Questionnaire (A1)

Question 4 Instructions: *Read choices out loud to participant. Mark the one best answer.*

4. I would like to ask you about your employment status. Are you now...

- ☐ regularly employed full time?
- ☐ employed part-time?
- ☐ employed seasonally?
- ☐ self-employed?
- ☐ unemployed/looking for work?
- ☐ unemployed/not looking for work?
- ☐ retired or disabled?
- ☐ other? If other, specify: ____________________
- ☐ *REF*

5. Are you currently married (civil or traditional) or living together with a man/woman as married?

- ☐ *yes, currently married*
- ☐ *yes, living with a man/woman*
- ☐ *no*
- ☐ *REF*

→ *If no or REF, skip to Question 7 Instructions.*

6. Is your husband/wife or partner living with you now or is he/she staying elsewhere?

- ☐ *living with me*
- ☐ *staying elsewhere*
- ☐ *REF*

Question 7 Instructions: *This question for women only. If participant is male, skip to Sexual Activity section.*

7. Are you currently pregnant? ☐ *yes* ☐ *no* ☐ *DK* ☐ *REF*

SEXUAL ACTIVITY

Instructions: *This section of the form addresses sexual behaviors and asks that the participant recall his/her sexual partners over the* ***past 6 months****.*

Interviewer reads:

Now I would like to ask you some questions about your recent sexual activity. I know these questions are sensitive and want to remind you that your answers are completely private. This means that they will not be shared with anyone outside of the study team. No one will know what particular answers you give. This form will not have your name anywhere on it. Instead, you will only be identified by a number. If we should come to any questions that you don’t want to answer, just let me know and we will go on to the next one.

Different people have different definitions of “sex” or “sexual intercourse.” For this study, when we say “sex” we mean:

- Vaginal sex, which is when a man puts his penis in a woman’s vagina.
- Anal sex, which is when a man puts his penis in another person’s anus.

Do you have any questions before continuing?

PTID

☐-☐☐☐-☐☐-☐-☐☐

Routine Questionnaire (A1)

8. How old were you when you had sexual intercourse for the very first time?

   age (years) ☐☐ ☐ *have never had sex* → *If have never had sex, skip to HIV Status section.* ☐ *REF*

9. In total, with how many different people have you had sexual intercourse in the last 6 months? It is okay to estimate the number if you do not remember exactly.

   number of partners ☐☐ → *If zero, skip to HIV Status section.* ☐ *REF*

10. With the ______ (*insert number of partners from question 9 or say "this or these" if 9 = REF*) sex partners that you have had in the last 6 months, how often did you use a condom when you had sexual intercourse?

    ☐ *sometimes* ☐ *always* ☐ *never* ☐ *DK* ☐ *REF*

Instructions: *Read* ***down*** *each column of the table (for each partner, one at a time),* ***not*** *across each row.*

Interviewer reads:

Now I would like to ask you more details about your most recent sex partners in the last 6 months. Please tell me about them starting with the most recent sex partner.

	Partner 1	☐ *no 2nd partner* *Skip to HIV Status section.* ***Partner 2***	☐ *no 3rd partner* *Skip to HIV Status section.* ***Partner 3***
11. First name, nickname, or marker of each partner	______ ☐ *REF*	______ ☐ *REF*	______ ☐ *REF*
12. Month/year sexual relationship began	*MMM* ☐☐☐ *yy* ☐☐ ☐ *REF*	*MMM* ☐☐☐ *yy* ☐☐ ☐ *REF*	*MMM* ☐☐☐ *yy* ☐☐ ☐ *REF*
13. Month/year sexual relationship ended *Interviewer: Record today's date if relationship has not ended.*	*MMM* ☐☐☐ *yy* ☐☐ ☐ *REF*	*MMM* ☐☐☐ *yy* ☐☐ ☐ *REF*	*MMM* ☐☐☐ *yy* ☐☐ ☐ *REF*
14. Partner's sex	☐ *male* ☐ *female* ☐ *REF*	☐ *male* ☐ *female* ☐ *REF*	☐ *male* ☐ *female* ☐ *REF*

PTID

☐-☐☐☐-☐☐-☐-☐☐

Routine Questionnaire (A1)

Instructions: *If response to Question 14 is female or REF, skip to 15. If response to Question 14 is "male", continue to Question 14a.*

	**Partner 1**	**Partner 2**	**Partner 3**
14a. Was his penis circumcised or uncircumcised?<br><br>*Interviewer: Show participant male circumcision drawings on Interview Card #1.*	☐ *circumcised*<br>☐ *uncircumcised*<br>☐ *became circumcised during relationship*<br>☐ *DK*<br>☐ *REF*	☐ *circumcised*<br>☐ *uncircumcised*<br>☐ *became circumcised during relationship*<br>☐ *DK*<br>☐ *REF*	☐ *circumcised*<br>☐ *uncircumcised*<br>☐ *became circumcised during relationship*<br>☐ *DK*<br>☐ *REF*
15. About how old was she/he the first time you had sex with her/him?	*years* ☐☐ ☐ *REF*	*years* ☐☐ ☐ *REF*	*years* ☐☐ ☐ *REF*

For the next question, I am going to ask you if your partner was a husband/wife, a regular partner, or a casual partner:

- By husband/wife we mean someone who you are married to or living with as if married.
- By regular partner we mean someone who you are NOT married to or living with as married, but who is a steady partner such as a girlfriend or boyfriend.
- By casual partner we mean someone who is NOT your spouse or a regular partner, but with whom you have had sex with in the last 6 months.

16. Keeping these definitions in mind, is this partner your spouse, a regular partner, or a casual partner?	☐ *husband/wife*<br>☐ *regular partner*<br>☐ *casual partner*<br>☐ *REF*	☐ *husband/wife*<br>☐ *regular partner*<br>☐ *casual partner*<br>☐ *REF*	☐ *husband/wife*<br>☐ *regular partner*<br>☐ *casual partner*<br>☐ *REF*
17. On approximately how many days did you have sex with him/her in the last 6 months?	☐ *1*<br>☐ *between 2–5*<br>☐ *between 6–10*<br>☐ *more than 10*<br>☐ *REF*	☐ *1*<br>☐ *between 2–5*<br>☐ *between 6–10*<br>☐ *more than 10*<br>☐ *REF*	☐ *1*<br>☐ *between 2–5*<br>☐ *between 6–10*<br>☐ *more than 10*<br>☐ *REF*

Instructions: *For questions 18–25, show participant Interview Card #2 to help them remember the response options: always, sometimes, or never.*

18. How often did you use a condom when you had sexual intercourse?	☐ *always*<br>☐ *sometimes*<br>☐ *never*<br>☐ *REF*	☐ *always*<br>☐ *sometimes*<br>☐ *never*<br>☐ *REF*	☐ *always*<br>☐ *sometimes*<br>☐ *never*<br>☐ *REF*

PTID ☐-☐☐☐-☐☐-☐-☐☐

Routine Questionnaire (A1)

	Partner 1	Partner 2	Partner 3
19. How often did you give or receive money or gifts so that you would have sex with this person?	☐ *always*<br>☐ *sometimes*<br>☐ *never*<br>☐ *REF*	☐ *always*<br>☐ *sometimes*<br>☐ *never*<br>☐ *REF*	☐ *always*<br>☐ *sometimes*<br>☐ *never*<br>☐ *REF*
20. Did you and your partner engage in *vaginal* sex in the last 6 months?	☐ *yes* ☐ *REF*<br>☐ *no*	☐ *yes* ☐ *REF*<br>☐ *no*	☐ *yes* ☐ *REF*<br>☐ *no*
		If no, skip to 22.	
21. How often did you and your partner use a condom when you had *vaginal* sex in the last 6 months?	☐ *always*<br>☐ *sometimes*<br>☐ *never*<br>☐ *REF*	☐ *always*<br>☐ *sometimes*<br>☐ *never*<br>☐ *REF*	☐ *always*<br>☐ *sometimes*<br>☐ *never*<br>☐ *REF*
22. Did you and your partner engage in *anal* sex in the last 6 months?	☐ *yes* ☐ *REF*<br>☐ *no*	☐ *yes* ☐ *REF*<br>☐ *no*	☐ *yes* ☐ *REF*<br>☐ *no*
		If no, skip to Questions 24–25 Instructions.	
23. How often did you and your partner use a condom when you had *anal* sex in the last 6 months?	☐ *always*<br>☐ *sometimes*<br>☐ *never*<br>☐ *REF*	☐ *always*<br>☐ *sometimes*<br>☐ *never*<br>☐ *REF*	☐ *always*<br>☐ *sometimes*<br>☐ *never*<br>☐ *REF*

Questions 24–25 Instructions: *Complete questions 24–25 for all male participants who had a male sex partner(s) in the past 6 months. All other participants, skip to question 26.*

	Partner 1	Partner 2	Partner 3
24. Did you and your partner have *anal* sex in the last 6 months?	☐ *yes* ☐ *REF*<br>☐ *no*	☐ *yes* ☐ *REF*<br>☐ *no*	☐ *yes* ☐ *REF*<br>☐ *no*
		If no, skip to 26.	
25. How often did you and your partner use a condom when you had *anal* sex in the last 6 months?	☐ *always*<br>☐ *sometimes*<br>☐ *never*<br>☐ *REF*	☐ *always*<br>☐ *sometimes*<br>☐ *never*<br>☐ *REF*	☐ *always*<br>☐ *sometimes*<br>☐ *never*<br>☐ *REF*

PTID ☐-☐☐☐-☐☐-☐-☐☐

Routine Questionnaire (A1)

	Partner 1	Partner 2	Partner 3
26. When you were having a sexual relationship with this partner, do you think that he/she was HIV positive?	☐ yes ☐ DK ☐ no ☐ REF	☐ yes ☐ DK ☐ no ☐ REF	☐ yes ☐ DK ☐ no ☐ REF
27. Do you think that this partner was taking ART for HIV/AIDS?	☐ yes ☐ DK ☐ no ☐ REF	☐ yes ☐ DK ☐ no ☐ REF	☐ yes ☐ DK ☐ no ☐ REF

HIV STATUS INFORMATION

Instructions: *This section of the form addresses prior HIV testing.*

Interviewer reads:
Now I would like to ask you some questions about HIV testing. Your answers are completely private. This form will not have your name anywhere on it; instead you will only be identified by a number.

28. Have you ever been tested to see if you have the AIDS virus?
☐ *yes* ☐ *no* ☐ *DK* ☐ *REF* → *If no, DK, or REF, skip to 34.*

29. How many times have you had an HIV test in your lifetime?
number of times ☐☐ ☐ *REF*

30. When was the last time you had an HIV test? Give best approximate date.
MMM ☐☐☐ *yy* ☐☐ *REF* ☐

31. Did you get the result of your last HIV test?
☐ *yes* ☐ *no* ☐ *DK* ☐ *REF* → *If DK or REF, skip to 34.*
If yes, skip to 33.

Question 32 Instructions: *Do not read. Record reason as described by participant.*

32. What are some of the reasons that you did not get your HIV test result?

☐ *I did not want to know/was afraid to know my test result*
☐ *provider did not give result to me*
☐ *had to get partner permission to test*
☐ *wanted to test with partner*
☐ *did not have time to wait for result*
☐ *other* ______________________

PTID

☐-☐☐☐-☐☐-☐-☐☐

Routine Questionnaire (A1)

Instructions: *If question 32 was answered, skip to HIV Prevention Exposure Information section..*

	positive	*negative*	*indeterminate*	*DK*	*REF*
33. I would like to ask you the result of your latest HIV test, but I want to remind you again that you should only answer the question if you feel comfortable. If you feel comfortable, could you tell me the result of your latest HIV test?	☐	☐	☐	☐	☐

	yes	*no*	*DK*	*REF*
34. Has a doctor or nurse ever told you that you should be taking ART to treat HIV (including during pregnancy)?	☐	☐	☐	☐
35. Are you currently taking ART to treat HIV?	☐	☐	☐	☐

HIV PREVENTION EXPOSURE INFORMATION

Instructions: *Read each question and mark yes or no, as appropriate. Each time a participant answers 'yes', ask the participant "What is the source of this information?" Read the list of sources of information aloud. Show participant Interviewer Card #4 with response categories. Use the key below to indicate the source(s) that correspond to the participant's answer(s). More than one response is acceptable.*

1 = Billboard
2 = Radio
3 = Television
4 = Community group/organization
5 = Health care provider
6 = Religions leader/organization
7 = Friend
8 = Family member
9 = Other

Interviewer reads:
Now I would like to ask you some questions about HIV prevention messages that you may have heard or seen in the past 6 months and how or where you heard or saw them. Please use this card to help you answer.

36. In the past 6 months, have you heard or seen any messages about the following topics related to HIV?

If yes, ask: What is the source of this information? *Mark all that apply.*

	yes	*no*	*REF*	1	2	3	4	5	6	7	8	9
36a. Get an HIV test to know your status.	☐	☐	☐	☐	☐	☐	☐	☐	☐	☐	☐	☐
36b. Reduce your number of sex partners.	☐	☐	☐	☐	☐	☐	☐	☐	☐	☐	☐	☐
36c. Use condoms every time you have sex.	☐	☐	☐	☐	☐	☐	☐	☐	☐	☐	☐	☐

PTID

□-□□□-□□-□-□□

Routine Questionnaire (A1)

				If yes, ask: What is the source of this information? *Mark all that apply.* 1 2 3 4 5 6 7 8 9
36d. Male circumcision for HIV prevention.	□	□	□	□ □ □ □ □ □ □ □ □
36e. ART is available in clinics to treat HIV.	□	□	□	□ □ □ □ □ □ □ □ □
36f. All pregnant women should get an HIV test.	□	□	□	□ □ □ □ □ □ □ □ □
36g. ART is available to prevent a mother from transmitting HIV to her baby.	□	□	□	□ □ □ □ □ □ □ □ □
36h. Other, specify: ____________ ____________	□	□	□	□ □ □ □ □ □ □ □ □

Instructions: *If the participant is female, skip to Final Statement section. If the participant is male, continue to Male Circumcision Status section.*

MALE CIRCUMCISION STATUS

Interviewer reads:
Now I would like to ask you about male circumcision. I am going to show you some drawings to help answer the questions. As a reminder, by male circumcision, I mean removal of the foreskin of the penis. Before we begin, do you have any questions?

Instructions: *Show participant male circumcision drawings on Interview Card #1.*

37. Based on these drawings, when you do NOT have an erection, would you say your penis is uncircumcised (more like the first drawing) or circumcised (more like the second drawing)?

circumcised □ *uncircumcised* □ *DK* □ *REF* □ → *If uncircumcised, DK, or REF, skip to Final Statement.*

38. When were you circumcised? *MMM* □□□ *yyyy* □□□□ □ *REF* → *If REF, skip to Final Statement.*

FINAL STATEMENT

Interviewer reads:
Thank you very much for your cooperation. The information you provided is very helpful and we appreciate your time and assistance. Do you have any final questions or comments that you would like to share with me?
