# Supplementary material for: HIV incidence, viremia, and the national response in Eswatini: Two sequential population-based surveys
Source: PLoS One. 2021 Dec 2;16(12):e0260892. doi: 10.1371/journal.pone.0260892 (PMC8639055; doi:10.1371/journal.pone.0260892)
Supplement: S2 File — (PDF) [file pone.0260892.s002.pdf]

SHIMS2

SWAZILAND HIV INCIDENCE MEASUREMENT SURVEY

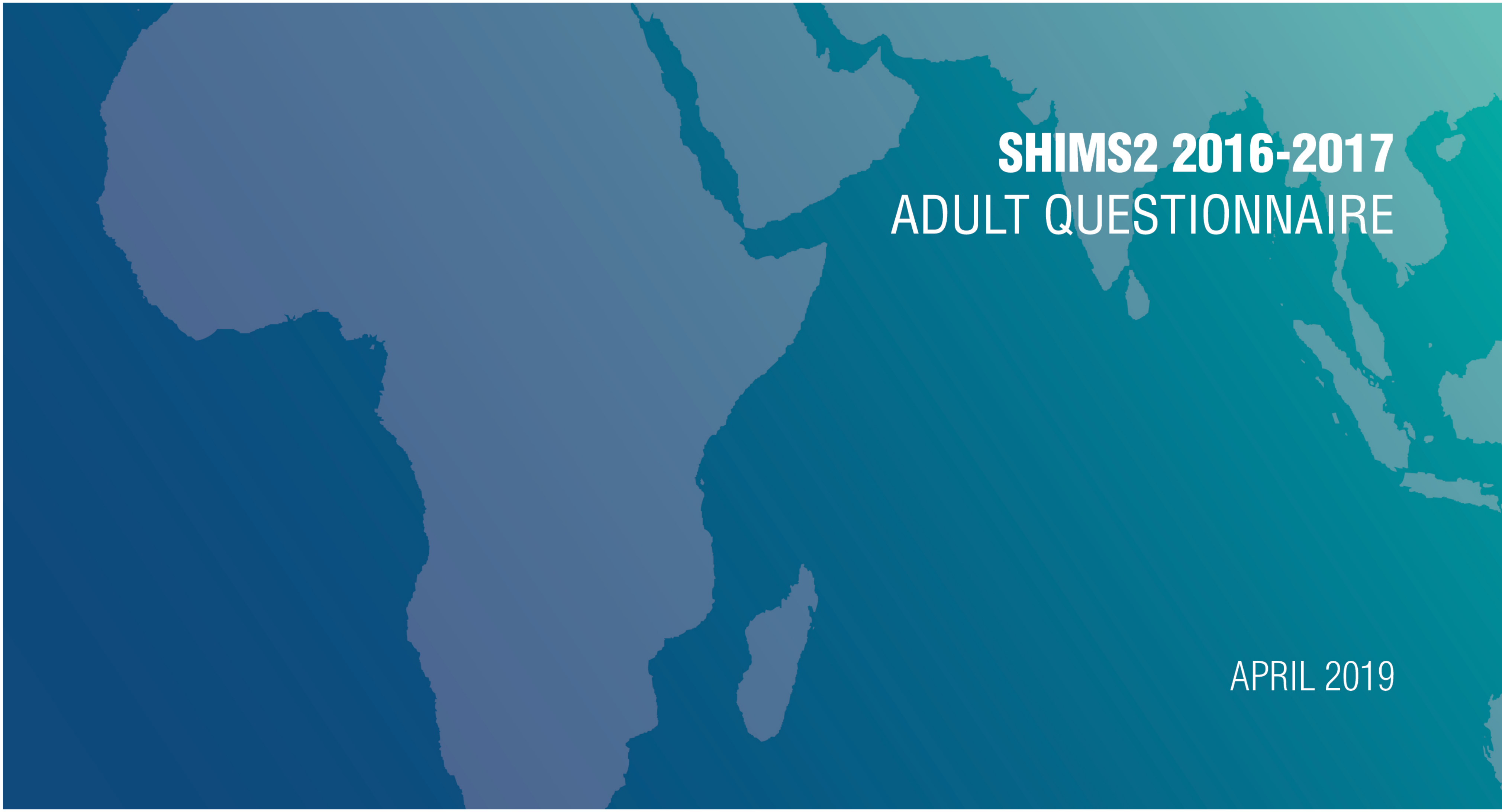

SHIMS2 2016-2017
ADULT QUESTIONNAIRE

APRIL 2019

The mark "CDC" is owned by the US Dept. of Health and Human Services and is used with permission. Use of this logo is not an endorsement by HHS or CDC of any particular product, service, or enterprise.
This project is supported by the U.S. President's Emergency Plan for AIDS Relief (PEPFAR) through CDC under the terms of cooperative agreement #U2GGH001226. The contents of this document do not necessarily represent the official position of the funding agencies.

NO.	VARIABLE	QUESTIONS	CODING LABELS	CODING VALUES	SKIP PATTERNS	SKIP TO
L1	lngvqx	DO NOT READ: LANGUAGE OF QUESTIONNAIRE	ENGLISH	1		
			SISWATI	2		
L2	lngvint	DO NOT READ: LANGUAGE OF INTERVIEW	ENGLISH	1	IF lngvint != 96	lngvnat
			SISWATI	2		
			OTHER (SPECIFY)	96		
L3	lngvnat	DO NOT READ: NATIVE LANGUAGE OF PARTICIPANT	ENGLISH	1	IF lngvnat != 96	trnsluse
			SISWATI	2		
			OTHER (SPECIFY)	96		
L4	trnsluse	DO NOT READ: TRANSLATOR USED	YES	1		
			NO	2		
MTITLE		**MODULE 1: RESPONDENT BACKGROUND**				
INSTR	Process variable removed from dataset.	Thank you for agreeing to participate in this survey. The first set of questions is about your life in general. Afterwards, we will move on to other topics.				
101	gender	Gender was previously given as ${bckggend_disp}*. If this is not correct, please review previously given answer in the Eligibility Form and update as needed.				
102	schlat	Have you ever attended school?	YES	1	IF schlat = 2,-8,-9	mont12away
			NO	2		
			DON'T KNOW	-8		
			REFUSED	-9		
103	schlcur	Are you enrolled in school?	YES	1		
			NO	2		
			DON'T KNOW	-8		
			REFUSED	-9		
104	schlhi	What is the highest level of school you attended: primary, secondary, or higher?	PRIMARY	1		
			SECONDARY	2		
			HIGH SCHOOL	3		
			TERTIARY	4		
			DON'T KNOW	-8		
			REFUSED	-9		
105	schcom	What is the highest grade/form that you have completed? MARK GRADE OR FORM, AS APPROPRIATE.	GRADE/FORM	integer		
			NURSERY/KINDERGARTEN	0		
			GRADE	1		
			FORM	2		
			DON'T KNOW	-8		
			REFUSED	-9		
106	mont12away	In the last 12 months, how many times have you been away from home for one or more nights?	NUMBER OF TRIPS	integer	IF mont12away = 0, -8,-9	work12mo
	mont12awaydk		DON'T KNOW	-8		
			REFUSED	-9		

NO.	VARIABLE	QUESTIONS	CODING LABELS	CODING VALUES	SKIP PATTERNS	SKIP TO
107	mo12away	In the last 12 months, have you been away from home for more than one month at a time?	YES	1		
			NO	2		
			DON'T KNOW	-8		
			REFUSED	-9		
108	work12mo	Have you done any work in the last 12 months for which you received a paycheck, cash or goods as payment?	YES	1	IF work12mo=2,-8,-9	[NEXT MODULE]
			NO	2		
			DON'T KNOW	-8		
			REFUSED	-9		
109A	work30days	Have you done any work in the last thirty days for which you received cash or goods as payment?	YES	1		
			NO	2		
			DON'T KNOW	-8		
			REFUSED	-9		
CONT		DO NOT READ: CONTINUE TO THE NEXT ITEM?	YES	1	IF item= 2	**INDIVIDUAL REFUSAL OR WITHDRAWAL**
		SELECT 'NO' ONLY IF THE PARTICIPANT HAS EXPRESSED HE/SHE DOES NOT WISH TO CONTINUE ON WITH THE SURVEY.	NO	2		
MTITLE		**MODULE 2: MARRIAGE**				
INSTR2		Now I would like to ask you about your current and previous relationships and/or marriages.				
201	evermar	Have you ever been married or lived together with a [man/woman] as if married?	YES	1	IF evermar = 2, -8, -9	[NEXT MODULE]
			NO	2		
			DON'T KNOW	-8		
			REFUSED	-9		
202	curmar	What is your marital status now: are you married, living together with someone as if married, widowed, divorced, or separated?	MARRIED	1	IF curmar = 3, 4, 5, -8, -9	[NEXT MODULE]
			LIVING TOGETHER	2		
			WIDOWED	3		
			DIVORCED	4		
			SEPARATED	5		
			DON'T KNOW	-8		
			REFUSED	-9		
INSTR		The next several questions are about your current spouse or partner(s).			If gender is Female and partner linked in roster	huslivew
					If gender is Female and no partner linked in roster	husotwif
203	numwif<br>numwifdk	Altogether, how many wives or partners do you have?	NUMBER OF WIVES OR PARTNERS<br>DON'T KNOW	integer<br>-8	If no linked partner in roster	npnyhstd

NO.	VARIABLE	QUESTIONS	CODING LABELS	CODING VALUES	SKIP PATTERNS	SKIP TO
			REFUSED	-9		
204	reviewwife	The Household Schedule listed [INSERT NUMBER OF REPORTED PARTNERS] household members as your wives/partners. Please review the list below. Are all of the listed household members your wives/partners who live in the household?	YES	1	IF reviewwife = 1	npyn
		[DISPLAY QXA1205_LIST]	NO	2		
group start						
205	Redacted	Is [HHRNAME**] your wife/partner?	YES	1	IF item = 2	npyn
			NO	2		
206	livehere	Does [HHRNAME**] live in the household?	YES	1		
			NO	2		
group end						
207	npyn	Do you have other wives/partner(s) that live with you in this household?	YES	1	IF npyn = 2	npnyhstd
			NO	2		
208	npnum	How many other wives/partners(s) live with you in this household?	NUMBER OF WIVES OR PARTNERS	integer	IF npnum = -8, -9	npnyhstd
	npnumdk		DON'T KNOW	-8		
			REFUSED	-9		
group start					# of Repeats = count in npnum	
209	Redacted	Please enter the name of your wife/partner that lives with you in this household.		text		
			DON'T KNOW	-8		
			REFUSED	-9		
group end						
210	npnyhstd	Do you have other wives/partner(s) that live with you in this homestead	YES	1	IF npnyhstd = 2	wifliveew
			NO	2		
211	npnumhstd	How many other wives/partners(s) live with you in this homestead?	YES	integer		
			DON'T KNOW	-8		
			REFUSED	-9		
212	wifliveew	How many wives or live-in partners do you have who live outside this homestead?		integer	ALL	[NEXT MODULE]
	wifliveewdk		DON'T KNOW	-8		
			REFUSED	-9		
	huslivew	Is your husband or partner living with you now or is he staying elsewhere?	LIVING TOGETHER	1	IF huslivew = 2 and count of household roster list of husbands/partners = 0	husotwif
			STAYING ELSEWHERE	2	IF huslivew = -8, -9 and no partner linked in roster	husotwif
			DON'T KNOW	-8		
			REFUSE TO ANSWER	-9		
213	Process variable removed from dataset.	The household schedule listed [QXA1205_LIST] as your husband/partner who is living here. Is that correct?	YES	1	IF item = 1, -8, -9	husotwif

NO.	VARIABLE	QUESTIONS	CODING LABELS	CODING VALUES	SKIP PATTERNS	SKIP TO
			NO	2		
			DON'T KNOW	-8		
			REFUSE TO ANSWER	-9		
214	husid	Please select the husband/partner that lives with you.	[LIST OF PERSONS on HH ROSTER]		IF husid != 96	husotwif
			NOT LISTED IN HOUSEHOLD	96		
215	Redacted	Please enter the name of your husband/partner that lives with you.		text		
			DON'T KNOW	-8		
			REFUSED	-9		
216	husotwif	Does your husband or partner have other wives or does he live with other women as if married?	YES	1	IF husotwif = 2, -8, -9	[NEXT MODULE]
			NO	2		
			DON'T KNOW	-8		
			REFUSE TO ANSWER	-9		
217	husnwif	Including yourself, in total, how many wives or live-in partners does your husband or partner have?	NUMBER OF WIVES OR LIVE-IN PARTNERS	integer		
	husnwifdk		DON'T KNOW	-8		
			REFUSE TO ANSWER	-9		
CONT		DO NOT READ: CONTINUE TO THE NEXT ITEM?	YES	1	IF item =2	**INDIVIDUAL REFUSAL OR WITHDRAWAL**
		SELECT 'NO' ONLY IF THE PARTICIPANT HAS EXPRESSED HE/SHE DOES NOT WISH TO CONTINUE ON WITH THE SURVEY.	NO	2		
MTITLE		**MODULE 3: REPRODUCTION**			IF (gender* = 1)	item 342
INSTR		Now I would like to ask you questions about your pregnancies and your children.				
301	pregnum	How many times have you been pregnant including a current pregnancy?	NUMBER OF TIME(S)	integer		
	pregnumdk	CODE '0' IF NONE.	DON'T KNOW	-8	IF pregnum = 0	item 342
			REFUSED	-9	IF pregnum = -8, -9	item 342
302	liveb	Have you ever had a pregnancy that resulted in a live birth?	YES	1	IF liveb = 2, -8, -9	item 342
		A live birth is when the baby shows signs of life, such as breathing, beating of the heart or movement.	NO	2		
			DON'T KNOW	-8		
			REFUSED	-9		
303	childa2012	How many live births have you had since the 1st of January, 2013?	NUMBER OF LIVE BIRTHS	integer	IF childa2012 = 0, -8, -9	item 342
	childa2012dk	DO NOT READ: CODE '0' IF NONE.	DON'T KNOW	-8		
			REFUSED	-9		
INSTR		Now I would like to ask you some questions about the last pregnancy that resulted in a live birth since the 1st of January, 2013.				
304	prgtwin	Did your last pregnancy result in birth to twins or more?	YES	1	IF prgtwin = 2, -8, -9	childlast
			NO	2	IF prgtwin = 1	pregnm
			DON'T KNOW	-8		

NO.	VARIABLE	QUESTIONS	CODING LABELS	CODING VALUES	SKIP PATTERNS	SKIP TO
			REFUSED	-9		
	pregnm	How many live children were born from your last pregnancy?	Number of children	integer		
group start						
305	Redacted	What is the name of the [BIRTHORDER*] born child from your last pregnancy that resulted in a live birth?<br>A live birth is when the baby shows signs of life, such as breathing, beating of the heart or movement.<br>DO NOT READ: IF THE CHILD WAS NOT NAMED BEFORE DEATH, INPUT BIRTH AND THE BIRTH ORDER NUMBER. FOR EXAMPLE, "BIRTH 1".		text		
group end						
306	redacted	What is the name of the child from your last pregnancy that resulted in a live birth?<br>A live birth is when the baby shows signs of life, such as breathing, beating of the heart or movement.<br>DO NOT READ: IF THE CHILD WAS NOT NAMED BEFORE DEATH, INPUT BIRTH 1.	NAME	text		
307	prgcare	When you were pregnant with [CHILDLAST/PRGTWINNAME*], did you visit a health facility for clinic visits for the pregnancy?	YES	1	IF prgcare = 1	item 309
			NO	2	IF prgcare = -8, -9	brthwhr
			DON'T KNOW	-8		
			REFUSED	-9		
308	pregncr	What is the <u>main</u> reason you did not visit a clinic for the pregnancy when you were pregnant with [CHILDLAST/PRGTWINNAME*]?	CLINIC WAS TOO FAR AWAY	1	IF pregncr != 96	brthwhr
			COULD NOT TAKE TIME OFF WORK/TOO BUSY	2		
			COULD NOT AFFORD TO PAY FOR THE VISIT	3		
			DID NOT TRUST THE CLINIC STAFF	4		
			RECEIVED CARE AT HOME	5		
			DID NOT WANT AN HIV TEST DONE	6		
			HUSBAND/FAMILY WOULD NOT LET ME GO	7		
			USED TRADITIONAL BIRTH ATTENDANT/HEALER	8		
			COST OF TRANSPORT	9		
			RELIGIOUS REASONS	10		
			OTHER (SPECIFY)	96		
			DON'T KNOW	-8		
			REFUSED	-9		

NO.	VARIABLE	QUESTIONS	CODING LABELS	CODING VALUES	SKIP PATTERNS	SKIP TO
INSTR3B		I will now be asking you questions on HIV testing. Please remember that your responses will be kept confidential and will not be shared with anyone else.				
309	hivtsbp	Have you ever tested for HIV before your pregnancy with [CHILDLAST/PRGTWINNAME*]?	YES	1	IF hivtsbp = 2, -8, -9	hivtopg
			NO	2		
			DON'T KNOW	-8		
			REFUSED	-9		
310	hivpsbp	Did you test positive for HIV before your pregnancy with [CHILDLAST/PRGTWINNAME*]?	YES	1	IF hivpsbp = 2, -8, -9	hivtopg
			NO	2		
			DON'T KNOW	-8		
			REFUSED	-9		
311	arvfvst	At the time of your first clinic visit for the pregnancy when you were pregnant with [CHILDLAST/PRGTWINNAME*], were you taking ARVs, that is, antiretroviral medications, to treat HIV?	YES	1	IF arvfvst = 1	brthwhr
			NO	2	IF arvfvst = 2, -8, -9	arvtkpg
			DON'T KNOW	-8		
			REFUSED	-9		
312	hivtopg	During any of your clinic visits for the pregnancy when you were pregnant with [CHILDLAST/PRGTWINNAME*], were you offered an HIV test?	YES	1		
			NO	2		
			DON'T KNOW	-8		
			REFUSED	-9		
313	hivtprg	Were you tested for HIV during any of your clinic visits for the pregnancy when you were pregnant with [CHILDLAST/PRGTWINNAME*]?	YES	1	IF hivtprg = 1	hivrtpg
			NO	2	IF hivtprg = -8, -9	brthwhr
			DON'T KNOW	-8		
			REFUSED	-9		
314	hivtsnr	What is the main reason you were not tested for HIV during clinic visits for the pregnancy with [CHILDLAST/PRGTWINNAME*]?	DID NOT WANT AN HIV TEST DONE / DID NOT WANT TO KNOW MY STATUS	1	hivtsnr != 96	brthwhr
			DID NOT RECEIVE PERMISSION FROM SPOUSE/FAMILY	2		
			AFRAID OTHERS WOULD KNOW ABOUT TEST RESULTS	3		
			DID NOT NEED TEST/LOW RISK	4		
			OTHER (SPECIFY)	96		
			DON'T KNOW	-8		
			REFUSED	-9		
315	hivrtpg	What was the result of your last HIV test during your pregnancy with [CHILDLAST/PRGTWINNAME*]?	POSITIVE	1	IF hivrtpg = 2, 3, 4, -8, -9	brthwhr
			NEGATIVE	2		

NO.	VARIABLE	QUESTIONS	CODING LABELS	CODING VALUES	SKIP PATTERNS	SKIP TO
			UNKNOWN/INDETERMINATE	3		
			DID NOT RECEIVE RESULTS	4		
			DON'T KNOW	-8		
			REFUSED	-9		
316	arvtkpg	Did you take ARVs during your pregnancy with [NAME] to stop [CHILDLAST/PRGTWINNAME*] from getting HIV?	YES	1	IF arvtkpg = 1, -8, -9	brthwhr
		DO NOT READ: SHOW ARV GRAPHIC IF PARTICIPANT IS UNSURE.	NO	2		
			DON'T KNOW	-8		
			REFUSED	-9		
317	arvnrpg	What was the main reason you did not take ARVs while you were pregnant with [CHILDLAST/PRGTWINNAME*]?	WAS NOT PRESCRIBED	1	IF arvnrpg !=96	brthwhr
			I FELT HEALTHY/NOT SICK	2		
			COST OF MEDICATIONS	3		
			COST OF TRANSPORT	4		
			RELIGIOUS REASONS	5		
			WAS TAKING TRADITIONAL MEDICATIONS	6		
			MEDICATIONS OUT OF STOCK	7		
			DID NOT WANT PEOPLE TO KNOW HIV STATUS	8		
			DID NOT RECEIVE PERMISSION FROM SPOUSE/FAMILY	9		
			OTHER (SPECIFY)	96		
			DON'T KNOW	-8		
			REFUSED	-9		
318	brthwhr	Where did you give birth to [CHILDLAST/PRGTWINNAME*]?	AT HOME	1	IF brthwhr = 1, 3, -8, -9	birthday
			AT A HEALTH FACILITY	2	IF brthwhr = 2	hivtobr
			IN TRANSIT	3		
			OTHER (SPECIFY)	96		
			DON'T KNOW	-8		
			REFUSED	-9		
319	hivtobr	Were you offered an HIV test during labor?	YES	1		
			NO	2		
			DON'T KNOW	-8		
			REFUSED	-9		
320	hivttlb	Did you test for HIV during labor?	YES	1	SKIP IF hivpsbp = 1 OR hivrtpg = 1	
			NO	2	IF hivttlb = 2, -8, -9	birthday
			DON'T KNOW	-8		
			REFUSED	-9		
321	hivrslr	What was the result of that test?	POSITIVE	1	SKIP IF hivpsbp = 1 OR hivrtpg = 1	
			NEGATIVE	2	IF hivrslr = 2, 3, 4, -8, -9	childbdate
			UNKNOWN/INDETERMINATE	3		
			DID NOT RECEIVE RESULTS	4		
			DON'T KNOW	-8		

NO.	VARIABLE	QUESTIONS	CODING LABELS	CODING VALUES	SKIP PATTERNS	SKIP TO
			REFUSED	-9		
322	offrarvs	During labor, were you offered ARVs to protect [NAME] against HIV?	YES	1	SKIP IF arvtkpg = 1	
		DO NOT READ: SHOW ARV GRAPHIC IF PARTICIPANT IS UNSURE.	NO	2		
			DON'T KNOW	-8		
			REFUSED	-9		
323	arvtklb	During labor, did you take ARVs to protect [CHILDLAST/PRGTWINNAME*] against HIV?	YES	1	SKIP IF arvtkpg = 1	
		DO NOT READ: SHOW ARV GRAPHIC IF PARTICIPANT IS UNSURE	NO	2	IF arvtklb = 2, -8, -9	birthday
			DON'T KNOW	-8		
			REFUSED	-9		
324	arvcntn	Did you continue to take the ARVs after delivery?	YES	1		
			NO	2	SKIP IF arvtkpg = 1	
			DON'T KNOW	-8		
			REFUSED	-9		
325	Redacted	When did you give birth to [CHILDLAST/PRGTWINNAME*]? Please give your best guess.	DAY	integer		
			DON'T KNOW DAY	-8		
			REFUSED DAY	-9		
	Redacted		MONTH	integer		
			DON'T KNOW MONTH	-8		
			REFUSED MONTH	-9		
	birthyr1-2		YEAR	integer		
			DON'T KNOW YEAR	-8		
			REFUSED YEAR	-9		
group start						
326	childlive1-2	Is [CHILDLAST/PRGTWINNAME*] still alive?	YES	1	IF childalive* = 1, -8, -9	childlivewith
			NO	2		
			DON'T KNOW	-8		
			REFUSE	-9		
327	deathageyr1-2	How old was[CHILDLAST/PRGTWINNAME*] when he/she died?	YEARS _____		deathageyr* > 0, -8, -9	childarv
		DO NOT READ: KEY '0' IF CHILD WAS LESS THAN ONE YEAR OLD.	DON'T KNOW	-8		
			REFUSED	-9		
328	deathagemo1-2	How old was [CHILDLAST/PRGTWINNAME*] in months when he/she died?	MONTHS ______		ALL	childarv
		DO NOT READ: KEY '0' IF CHILD WAS LESS THAN ONE MONTH OLD.	DON'T KNOW	-8		
			REFUSED	-9		
329	childlivewith1-2	Is [CHILDLAST/PRGTWINNAME*] living with you?	YES	1	IF childlivewith* = 2	childarv
			NO	2		

NO.	VARIABLE	QUESTIONS	CODING LABELS	CODING VALUES	SKIP PATTERNS	SKIP TO
330	hhlnchild1-2	DO NOT READ: RECORD HOUSEHOLD LINE NUMBER OF CHILD<br><br>RECORD ‘0’ IF CHILD NOT LISTED IN HOUSEHOLD	HOUSEHOLD LINE NUMBER	integer		
331	childarv1-2	Did [CHILDLAST/PRGTWINNAME*] take any ARVs to stop [him/her] from getting HIV infection? This would be before [CHILDLAST/PRGTWINNAME*]’s first HIV test.	YES	1	IF childarv* = 2, -8, -9	childbac*
		DO NOT READ: SHOW ARV GRAPHIC IF PARTICIPANT IS UNSURE.	NO	2		
			DON’T KNOW	-8		
			REFUSE	-9		
332	childarvt1-2	For how long did [CHILDLAST/PRGTWINNAME*] take the ARVs to stop him/her from getting HIV?	WEEKS	integer		
	childarvdurnumw1-2 / childarvdurnumm1-2	CODE ‘0’ IF LESS THAN ONE WEEK.	MONTHS	integer		
		ONLY ONE OPTION MAY BE SELECTED. FOR EXAMPLE, ANSWER ONLY IN WEEKS OR IN MONTHS.	ARVS TAKEN ONCE	96		
			STILL TAKING ARVS	97		
			DON’T KNOW	-8		
			REFUSED	-9		
333	childbac1-2	Did [CHILDLAST/PRGTWINNAME*] take Bactrim or cotrimoxazole? This would be before [CHILDLAST/PRGTWINNAME*]’s first HIV test.	YES	1	IF childbac* = 2, 3, -8, -9	childbrstfd*
		Bactrim or cotrimoxazole is a medicine recommended for people with HIV, even if they have not started treatment for HIV. It helps prevent certain infections but it is not a treatment for HIV.	NO, DID NOT TAKE COTRIM	2		
		DO NOT READ: SHOW COTRIMOXAZOLE GRAPHIC IF PARTICIPANT IS UNSURE.	NO, CHILD NOT ALIVE	3		
			DON’T KNOW	-8		
			REFUSED	-9		
334	childbact1-2	For how long did [CHILDLAST/PRGTWINNAME*] take Bactrim or cotrimoxazole?	WEEKS	integer		
	childscotrimdurnumw1-2 / childscotrimdurnumm1-2	ONLY ONE OPTION MAY BE SELECTED. FOR EXAMPLE, ANSWER ONLY IN WEEKS OR IN MONTHS.	MONTHS	integer		
		CODE ‘0’ IF LESS THAN ONE WEEK.	STILL TAKING COTRIMOXOZOLE	97		
			DON’T KNOW	-8		
			REFUSED	-9		
335	childbrstfd1-2	Did you ever breastfeed [CHILDLAST/PRGTWINNAME*]?	YES	1	IF childbrstfd* = 2, 3, -8, -9	item 339*
			NO, NEVER BREASTFED	2		
			NO, CHILD NOT ALIVE	3		
			DON’T KNOW	-8		
			REFUSED	-9		
337	childbrstfddur1-2	For how long did you breastfeed [CHILDLAST/PRGTWINNAME*]?	WEEKS	integer		
	childbrstfddurnum1-2	DO NOT READ: ONLY ONE OPTION MAY BE SELECTED. FOR EXAMPLE, ANSWER ONLY IN WEEKS OR IN MONTHS.	MONTHS	integer		

NO.	VARIABLE	QUESTIONS	CODING LABELS	CODING VALUES	SKIP PATTERNS	SKIP TO
		CODE ‘0” WEEKS IF LESS THAN 1 WEEK.	STILL BREASTFEEDING<br>DON’T KNOW<br>REFUSED	96<br>-8<br>-9		
338	childbfcont1-2	Did you continue taking ARVs while you were breastfeeding [CHILDLAST/PRGTWINNAME*]?	YES<br>NO<br>DON’T KNOW<br>REFUSED	1<br>2<br>-8<br>-9	SKIP IF arvfvst = 1 OR arvtkpg = 1	
339	Process variable removed from dataset.	Thank you for the information regarding [CHILDLAST/PRGTWINNAME*].			IF prgtwin = 1	RETURN TO childlive* FOR EACH VALUE OF prgtwinname*
group end						
INSTR		I will now ask about current pregnancies.				
340	pregnant	Are you pregnant now?	YES<br>NO<br>DON’T KNOW/UNSURE<br>REFUSED	1<br>2<br>-8<br>-9	IF pregnant = 2,-8,-9	item 335
341	pregmonths	How many months pregnant are you?<br>DO NOT READ: SHOW GRAPHIC OF PREGNANCY IF NOT SURE.	MONTHS<br>DON’T KNOW / UNSURE<br>REFUSED	integer<br>-8<br>-9	ALL	[NEXT MODULE]
INSTR		I will now ask you about family planning.				
342	avoidpreg	Are you or your partner currently doing something or using any method to delay or avoid getting pregnant?	YES<br>NO<br>DON’T KNOW<br>REFUSED	1<br>2<br>-8<br>-9	IF avoidpreg = 2, -8, -9	[NEXT MODULE]
343	cmethod_a<br>cmethod_b<br>cmethod_c<br>cmethod_d<br>cmethod_e<br>cmethod_f<br>cmethod_g<br>cmethod_h<br>cmethod_i<br>cmethod_j<br>cmethod_k<br>cmethod_x<br>cmethod_y<br>cmethod_z	Which method are you or your partner using?<br>DO NOT READ: SELECT ALL THAT APPLY.	FEMALE STERILIZATION<br>MALE STERILIZATION<br>PILL<br>IUD/”COIL”<br>INJECTIONS<br>IMPLANT<br>CONDOM<br>FEMALE CONDOM<br>RHYTHM/NATURAL METHODS<br>WITHDRAWAL<br>NOT HAVING SEX<br>OTHER (SPECIFY)<br>DON’T KNOW<br>REFUSED	A<br>B<br>C<br>D<br>E<br>F<br>G<br>H<br>I<br>J<br>K<br>X<br>Y<br>Z	IF cmethod_x != X	[NEXT MODULE]
CONT		DO NOT READ: CONTINUE TO THE NEXT ITEM?<br>SELECT 'NO' ONLY IF THE PARTICIPANT HAS EXPRESSED HE/SHE DOES NOT WISH TO CONTINUE ON WITH THE SURVEY.	YES<br>NO	1<br>2	IF item = 2	**INDIVIDUAL REFUSAL OR WITHDRAWAL**

NO.	VARIABLE	QUESTIONS	CODING LABELS	CODING VALUES	SKIP PATTERNS	SKIP TO
MTITLE		**MODULE 4: CHILDREN**				
INSTR		I am going to ask you a number of questions about your children regarding their health and where they get their health services. We will ask you about these children: LIST OF CHILDREN < 14 ASSIGNED TO [INNAME]				
group start						
401	Process variable removed from dataset.	Now I am going to ask you questions for [CHILD*].				
402	age	How old is [CHILD*] in years?	AGE IN YEARS	integer	IF age>=1 & age<=5	item 403a
		IF [CHILD*] IS LESS THAN 1 YEAR OLD, KEY 0 HERE AND KEY AGE IN MONTHS ON NEXT SCREEN.	DON'T KNOW	-8	IF age > 5	gender
			REFUSED	-9	IF age <1	agem
403	agem	How old is [CHILD*] in months?	AGE IN MONTHS	integer	ALL	gender
403A	Process variable removed from dataset.	You said that [CHILD*] was [KIDAGEY*]. How many months over [KIDAGEY*] is [CHILD*]?	MONTHS OVER	integer	ALL	gender
404	gender	Is [CHILD*] a boy or girl?	BOY	1		
			GIRL	2		
			DON'T KNOW	-8		
			REFUSED	-9		
405	ch_kidenroll	Is [CHILD*] enrolled in school?	YES	1	IF ch_kidenroll =3, -8, -9	ch_kidenrlstyr
			NO, CURRENTLY NOT IN SCHOOL	2		
			NO, TOO YOUNG TO BE IN SCHOOL	3		
			DON'T KNOW	-8		
			REFUSED	-9		
406	ch_kidhighlvl	What is the highest level of school [CHILD*] has attended: primary, secondary, or high school?	PRIMARY	1	IF ch_kidhighlvl = -8, -9	ch_kidenrlstyr
			SECONDARY	2	IF ch_kidhighlvl = 96	ch_kidcrcm
			HIGH SCHOOL	3		
			NEVER ATTENDED SCHOOL	96		
			DON'T KNOW	-8		
			REFUSED	-9		
407	ch_kidclass ch_kidclassunit	What grade/form is [CHILD*] in now?	GRADE/FORM	integer	SKIP IF ch_kidenroll = 2	
			NURSERY/KINDERGARTEN	0		
			GRADE	1		
			FORM	2		
			DON'T KNOW	-8		
			REFUSED	-9		
408	ch_kidenrlstyr	Was [CHILD*] enrolled in school during the previous school year?	YES	1	IF ch_kidenrlstyr = 2, -8, -9	ch_kidcrcm
			NO	2		
			DON'T KNOW	-8		
			REFUSED	-9		

NO.	VARIABLE	QUESTIONS	CODING LABELS	CODING VALUES	SKIP PATTERNS	SKIP TO
409	ch_kidclasslstyr	What grade/form was [CHILD*] during the previous school year?	GRADE/FORM	integer		
	ch_kidclasslstyrunit		NURSERY/KINDERGARTEN	0		
			GRADE	1		
			FORM	2		
			DON'T KNOW	-8		
			REFUSED	-9		
410	ch_kidcrcm	Is [CHILD*] circumcised?	YES	1	SKIP IF gender = 2	
		Circumcision is the complete removal of the foreskin from the penis. I have a picture to show you what a completely circumcised penis looks like.	NO	2	IF ch_kidcrcm = 2, -8, -9	ch_kidhivtestevr
		DO NOT READ: SHOW GRAPHIC	DON'T KNOW	-8		
			REFUSED	-9		
411	ch_kidcrcmprt	Who circumcised [CHILD*]?	DOCTOR, CLINICAL OFFICER, OR NURSE	1	SKIP IF gender = 2	
			TRADITIONAL PRACTITIONER / CIRCUMCISER	2	IF ch_kidcrcmprt != 96	ch_kidhivtestevr
			MIDWIFE	3		
			OTHER (SPECIFY)	96		
			DON'T KNOW	-8		
			REFUSE TO ANSWER	-9		
412	ch_kidhivtestevr	Has [CHILD*] ever been tested for HIV?	YES	1	IF ch_kidhivtestevr = 1	ch_kidhivtestm/ch_kidhivtesty
			NO	2	IF ch_kidhivtestevr = -8, -9	ch_kidvisttbclin
			DON'T KNOW	-8		
			REFUSED	-9		
413	ch_kidhivwhynever_a	Why has [CHILD*] never been tested for HIV?	DON'T KNOW WHERE TO TEST	A	ch_kidhivwhynever_x != X	ch_kidvisttbclin
	ch_kidhivwhynever_b	DO NOT READ: SELECT ALL THAT APPLY.	TEST COSTS TOO MUCH	B		
	ch_kidhivwhynever_c		TRANSPORT COSTS TOO MUCH	C		
	ch_kidhivwhynever_d		TOO FAR AWAY	D		
	ch_kidhivwhynever_e		AFRAID OTHERS WILL KNOW ABOUT TEST RESULTS	E		
	ch_kidhivwhynever_f		DON'T NEED TEST/LOW RISK	F		
	ch_kidhivwhynever_g		DID NOT RECEIVE PERMISSION FROM SPOUSE/FAMILY	G		
	ch_kidhivwhynever_h		AFRAID SPOUSE/PARTNER/FAMILY WILL KNOW RESULTS	H		
	ch_kidhivwhynever_i		DON'T WANT TO KNOW CHILD HAS HIV	I		
	ch_kidhivwhynever_j		CANNOT GET TREATMENT FOR HIV	J		
	ch_kidhivwhynever_k		TEST KITS NOT AVAILABLE	K		
	ch_kidhivwhynever_l		RELIGIOUS REASONS	L		
	ch_kidhivwhynever_x		OTHER (SPECIFY)	X		

NO.	VARIABLE	QUESTIONS	CODING LABELS	CODING VALUES	SKIP PATTERNS	SKIP TO
	ch_kidhivwhynever_y		DON'T KNOW	Y		
	ch_kidhivwhynever_z		REFUSED	Z		
414	ch_kidhivtestm	What month and year was [CHILD*]'s most recent HIV test done?	MONTH	date		
			DON'T KNOW MONTH	-8		
			REFUSED MONTH	-9		
	ch_kidhivtesty		YEARS	date		
			DON'T KNOW YEAR	-8		
			REFUSED YEAR	-9		
415	ch_kidhivlastresult	What was [CHILD*]'s last HIV test result?	POSITIVE	1	IF ch_kidhivlastresult = 2, 3, 4, -8, -9	ch_kidvisttbclin
			NEGATIVE	2		
			UNKNOWN/INDETERMINATE	3		
			DID NOT RECEIVE RESULTS	4		
			DON'T KNOW	-8		
			REFUSED	-9		
416	ch_kidtldpos	Has [CHILD*] been told (or know) that he/she is HIV positive?	YES	1	IF ch_kidtldpos = 1, -8, -9	ch_kidlposm/ch_kidlposy
			NO	2		
			DON'T KNOW	-8		
			REFUSED	-9		
417	ch_kidnttldposrsn	Why has [CHILD*] not been told they are HIV positive?	CHILD IS TOO YOUNG	1	IF ch_kidnttldposrsn != 96	ch_kidlposm/ch_kidlposy
			CHILD IS DEVELOPMENTALLY CHALLENGED	2		
			PARENT/LEGAL GUARDIAN FEARS TALKING TO CHILD ABOUT HIV	3		
			OTHER (SPECIFY)	96		
			DON'T KNOW	-8		
			REFUSED	-9		
418	ch_kidlposm	What was the month and year of [CHILD*]'s first HIV positive test result? Please give your best guess.	MONTH	date		
		This will be the very first HIV positive test result that you have received.	DON'T KNOW MONTH	-8		
		DO NOT READ: PROBE TO VERIFY DATE.	REFUSED MONTH	-9		
	ch_kidlposy		YEARS	date		
			DON'T KNOW YEAR	-8		
			REFUSED YEAR	-9		
419	ch_kidhivcare	Has [CHILD*] ever received HIV care and treatment from a doctor, clinical officer or nurse?	YES	1	IF ch_kidhivcare = 1	ch_kidhivcarefirstm/ch_kidhivcarefirsty
			NO	2	IF ch_kidhivcare = -8, -9	ch_kidcd4
			DON'T KNOW	-8		
			REFUSED	-9		

NO.	VARIABLE	QUESTIONS	CODING LABELS	CODING VALUES	SKIP PATTERNS	SKIP TO
420	ch_kidhivcarenvr	What is the main reason why [CHILD*] has never seen a doctor, clinical officer, or nurse for HIV care and treatment?	FACILITY IS TOO FAR AWAY	1	ch_kidhivcarenvr != 96	ch_kidcd4
			I DON'T KNOW WHERE TO GET HIV MEDICAL CARE FOR CHILD	2		
			COST OF CARE	3		
			COST OF TRANSPORT	4		
			I DON'T THINK CHILD NEEDS IT, HE/SHE IS NOT SICK	5		
			I FEAR PEOPLE WILL KNOW THAT CHILD HAS HIV IF I TAKE HIM/HER TO A CLINIC	6		
			RELIGIOUS REASONS	7		
			CHILD IS TAKING TRADITIONAL MEDICINE	8		
			CHILD HAS NOT BEEN TOLD THEY NEED HIV CARE	9		
			OTHER (SPECIFY)	96		
			DON'T KNOW	-8		
			REFUSED	-9		
421	ch_kidhivcarefirstm	What month and year did [CHILD*] first see a doctor, clinical officer or nurse for HIV care and treatment?	MONTH	date		
		DO NOT READ: PROBE TO VERIFY DATE.	DON'T KNOW MONTH	-8		
			REFUSED MONTH	-9		
	ch_kidhivcarefirsty		YEAR	date		
			DON'T KNOW YEAR	-8		
			REFUSED	-9		
422	ch_kidhivcarelastm	What month and year did [CHILD*] last see a doctor or nurse for HIV care and treatment?	MONTH	date	IF CURRENT DATE - ch_kidhivcarelastm/ch_kidhivcarelasty =< 6 MONTHS	ch_kidcd4
			DON'T KNOW MONTH	-8	IF ch_kidhivcarelastm/ch_kidhivcarelasty = -8, -9	ch_kidcd4
			REFUSED MONTH	-9		
	ch_kidhivcarelasty		YEAR	date		
			DON'T KNOW YEAR	-8		
			REFUSED	-9		
423	ch_kidhivnot6rsn	What is the main reason for [CHILD*] not seeing a doctor, clinical officer or nurse for HIV care and treatment for more than 6 months?	FACILITY IS TOO FAR AWAY	1	IF ch_kidhivnot6rsn != 96	ch_kidcd4
			I DON'T KNOW WHERE TO GET HIV MEDICAL CARE FOR CHILD	2		
			COST OF CARE	3		
			COST OF TRANSPORT	4		

NO.	VARIABLE	QUESTIONS	CODING LABELS	CODING VALUES	SKIP PATTERNS	SKIP TO
			I DON'T THINK CHILD NEEDS IT, HE/SHE IS NOT SICK	5		
			I FEAR PEOPLE WILL KNOW THAT CHILD HAS HIV IF I TAKE HIM/HER TO A CLINIC	6		
			RELIGIOUS REASONS	7		
			CHILD IS TAKING TRADITIONAL MEDICINE	8		
			NO APPOINTMENT SCHEDULED/DID NOT MISS MOST RECENT APPOINTMENT	9		
			OTHER (SPECIFY)	96		
			DON'T KNOW	-8		
			REFUSED	-9		
424	ch_kidcd4	Has [CHILD*] ever had a CD4 count test?	YES	1	IF ch_kidcd4 = 2, -8, -9 AND ch_kidhivcare = 1	ch_kidarvs
		The CD4 count tells you how sick you are with HIV.	NO	2	IF ch_kidcd4 = 2, -8, -9 AND ch_kidhivcare = 2, -8, -9	ch_kidseptrin
			DON'T KNOW	-8		
			REFUSED	-9		
425	ch_kidcd4lastm	What month and year was [CHILD*] last tested for his/her CD4 count?	MONTH	date	IF ch_kidcd4lastm = -8, -9	ch_kidarvs
			DON'T KNOW MONTH	-8	IF ch_kidcd4lasty = -8, -9	ch_kidarvs
			REFUSED MONTH	-9		
	ch_kidcd4lasty		YEAR	date		
			DON'T KNOW YEAR	-8		
			REFUSED YEAR	-9		
426	ch_kidarvs	Has [CHILD*] ever taken ARVs, that is, antiretroviral medications, to treat his/her HIV infection?	YES	1	IF ch_kidarvs = 1	ch_kidarvsfirsty/ch_kidarvsfirstm
		DO NOT READ: SHOW AID IF PARTICIPANT IS UNSURE.	NO	2	IF ch_kidarvs = -8, -9	ch_kidseptrin
			DON'T KNOW	-8		
			REFUSED	-9		
427	ch_kidarvsnvrrsn	What is the main reason [CHILD*] has never taken ARVs?	CHILD IS NOT ELIGIBLE FOR TREATMENT	1	IF ch_kidarvsnvrrsn != 96	ch_kidseptrin
			HEALTH CARE PROVIDER DID NOT PRESCRIBE	2		
			HIV MEDICINES NOT AVAILABLE	3		
			DO NOT THINK CHILD NEEDS IT, HE/SHE IS NOT SICK	4		
			COST OF MEDICATIONS	5		
			COST OF TRANSPORT	6		
			RELIGIOUS REASONS	7		
			CHILD IS TAKING TRADITIONAL MEDICATIONS	8		

NO.	VARIABLE	QUESTIONS	CODING LABELS	CODING VALUES	SKIP PATTERNS	SKIP TO
			CHILD HAS NOT BEEN TOLD THEY NEED ARVs	9		
			OTHER (SPECIFY)	96		
			DON'T KNOW	-8		
			REFUSED	-9		
428	ch_kidarvsfirstm	What month and year did [CHILD*] first start taking ARVs?	MONTH	date		
		[DO NOT READ]: FOR DO NOT KNOW: PROBE TO VERIFY DATE.	DON'T KNOW MONTH	-8		
			REFUSED MONTH	-9		
	ch_kidarvsfirsty		YEAR	date		
			DON'T KNOW YEAR	-8		
			REFUSED YEAR	-9		
429	ch_kidarvsnow	Is [CHILD*] currently taking ARVs, that is, antiretroviral medications?	YES	1		
		By currently, I mean that [CHILD*] may have missed some doses but [CHILD*] is still taking ARVs.	NO	2	IF ch_kidarvsnow = 1, -8, -9	ch_kidseptrin
			DON'T KNOW	-8		
			REFUSED	-9		
430	ch_kidarvsnotrsn	Can you tell me the main reason why [CHILD*] is not currently taking ARVs?	I HAVE TROUBLE GIVING CHILD A TABLET EVERYDAY	1	IF ch_kidarvsnotrsn != 96	ch_kidseptrin
			CHILD HAD SIDE EFFECTS/RASH	2		
			FACILITY/PHARMACY TOO FAR AWAY TO GET MEDICATION REGULARLY	3		
			COST OF MEDICATIONS	4		
			COST OF TRANSPORT	5		
			CHILD IS HEALTHY, HE/SHE IS NOT SICK	6		
			FACILITY WAS OUT OF STOCK	7		
			RELIGIOUS REASONS	8		
			CHILD IS TAKING TRADITIONAL MEDICATIONS	9		
			OTHER (SPECIFY)	96		
			DON'T KNOW	-8		
			REFUSED	-9		
431	ch_kidseptrin	Is [CHILD*] currently taking Bactrim, Septrin or cotrimoxazole?	YES	1		
		Bactrim, Septrin or cotrimoxazole is a medicine recommended for people with HIV, even if they have not started treatment for HIV. It helps prevent certain infections but it is not treatment for HIV.	NO	2		
		By currently, I mean that [CHILD*] may have missed some doses but is still taking Bactrim, Septrin or cotrimoxazole.	I DON'T KNOW WHAT IT IS	3		
		DO NOT READ: SHOW AID IF PARTICIPANT IS UNSURE	REFUSED	-9		

NO.	VARIABLE	QUESTIONS	CODING LABELS	CODING VALUES	SKIP PATTERNS	SKIP TO
	ch_kidvisttbclin	Has [CHILD*] ever visited a health facility or TB clinic for TB diagnosis or treatment?	YES	1	IF ch_kidvisttbclin = 2, -8, -9	item 433
			NO	2		
			DON'T KNOW	-8		
			REFUSED	-9		
	ch_kiddiagtb	Have you ever been told by a doctor, clinical officer or nurse that [CHILD*] had TB?	YES	1	IF ch_kiddiagtb = 2, -8, -9	item 433
			NO	2		
			DON'T KNOW	-8		
			REFUSED	-9		
	ch_kidtrttb	Was [CHILD*] ever treated for TB?	YES	1	IF ch_kidtrttb = 2, -8, -9	item 433
			NO	2		
			DON'T KNOW	-8		
			REFUSED	-9		
	ch_kidtrttcurr	Is [CHILD*] currently on treatment for TB?	YES	1		
			NO	2		
			DON'T KNOW	-8		
			REFUSED	-9		
	ch_kidtrttb6motrt	The last time [CHILD*] was treated for TB, did [CHILD*] complete at least 6 months of treatment?	YES	1		
			NO, THE MEDICINE WAS STOPPED IN LESS THAN 6 MONTHS	2		
			NO, [NAME] IS STILL ON TREATMENT	3		
			DON'T KNOW	-8		
			REFUSED	-9		
433	Process variable removed from dataset.	Thank you for the information about [CHILD*].			IF ADDITIONAL CHILD ON ROSTER	RETURN TO START OF MODULE 4
group end						
CONT		DO NOT READ: CONTINUE TO THE NEXT ITEM?	YES	1	IF item = 2	**INDIVIDUAL REFUSAL OR WITHDRAWAL**
		SELECT 'NO' ONLY IF THE PARTICIPANT HAS EXPRESSED HE/SHE DOES NOT WISH TO CONTINUE ON WITH THE SURVEY.	NO	2		
MTITLE		**MODULE 5: MALE CIRCUMCISION**			Skip module if female	
INSTR		I will be asking a few questions about circumcision. Circumcision is the complete removal of the foreskin from the penis. I have a picture to show you what a completely circumcised penis looks like.<br>DO NOT READ: SHOW AID				
501	mcriskr	Does male circumcision alone reduce the risk, or chance, of a man getting HIV completely, somewhat or not at all?	PROTECTS COMPLETELY	1		
			PROTECTS SOMEWHAT	2		
			NOT AT ALL	3		

NO.	VARIABLE	QUESTIONS	CODING LABELS	CODING VALUES	SKIP PATTERNS	SKIP TO
			DON'T KNOW	-8		
			REFUSED	-9		
502	mccndms	Do you agree or disagree with the following statement: Men who are circumcised do not need to use condoms to protect themselves from HIV.	STRONGLY AGREE	1		
			AGREE	2		
			DISAGREE	3		
			STRONGLY DISAGREE	4		
			DON'T KNOW	-8		
			REFUSED	-9		
503	mchiv	Do you agree or disagree with the following statement: Men who are circumcised can have multiple sexual partners and not be at risk for HIV.	STRONGLY AGREE	1		
			AGREE	2		
			DISAGREE	3		
			STRONGLY DISAGREE	4		
			DON'T KNOW	-8		
			REFUSED	-9		
504	mcstatus	Some men are uncomfortable talking about circumcision but it is important for us to have this information. Some men are circumcised and others are not. Are you circumcised?	YES	1	IF mcstatus = 1	mcage
			NO	2	IF mcstatus = -8, -9	[NEXT MODULE]
			DON'T KNOW	-8		
			REFUSED	-9		
505	mcplans	Are you planning to get circumcised?	YES	1	IF mcplans = 1, -8, -9	[NEXT MODULE]
			NO	2		
			DON'T KNOW	-8		
			REFUSED	-9		
506	mcnorsn_a	There may be different reasons why some men are circumcised and others are not. What are your reasons for not getting circumcised?	RESPONDENT OPPOSED	A	IF mcnorsn != 96	[NEXT MODULE]
	mcnorsn_b	SELECT ALL THAT APPLY.	PARTNER OPPOSED	B		
	mcnorsn_c		OTHERS OPPOSED	C		
	mcnorsn_d		RELIGIOUS PROHIBITION	D		
	mcnorsn_e		NO KNOWLEDGE ABOUT PROCEDURE UTILITY	E		
	mcnorsn_f		NO KNOWLEDGE SITE THAT PERFORMS PROCEDURE	F		
	mcnorsn_g		HEALTH CONCERNS	G		
	mcnorsn_h		FEAR OF SIDE EFFECTS	H		
	mcnorsn_i		LACK OF ACCESS TO FACILITY	I		
	mcnorsn_j		COSTS TOO MUCH	J		
	mcnorsn_k		INCONVENIENT	K		
	mcnorsn_l		INTERFERES WITH BODY'S NORMAL FUNCTIONING	L		

NO.	VARIABLE	QUESTIONS	CODING LABELS	CODING VALUES	SKIP PATTERNS	SKIP TO
	mcnorsn_x		OTHER (SPECIFY)	X		
	mcnorsn_y		DON'T KNOW	Y		
	mcnorsn_z		REFUSED	Z		
507	mcage	How old were you when you were circumcised? Please give your best guess.	AGE IN YEARS	integer		
	mcagedk	DO NOT READ: IF LESS THAN ONE YEAR, CODE '0'	DON'T KNOW	-8		
			REFUSED	-9		
508	mcwho	Who did the circumcision?	DOCTOR, CLINICAL OFFICER, OR NURSE	1	IF mcwho != 96	mcyesrn_a - z
			TRADITIONAL PRACTITIONER / CIRCUMCISER	2		
			MIDWIFE	3		
			OTHER (SPECIFY)	96		
			DON'T KNOW	-8		
			REFUSED	-9		
509	mcyesrn_a	There may be different reasons why some men are circumcised and others are not. What are your reasons for getting circumcised?	RELIGION	A	IF mcyesrn_x != X	[NEXT MODULE]
	mcyesrn_b		PREVENT HIV	B		
	mcyesrn_c	DO NOT READ: SELECT ALL THAT APPLY.	DON'T HAVE TO USE A CONDOM	C		
	mcyesrn_d		HYGIENE	D		
	mcyesrn_e		MY PARTNER ASKED ME TO	E		
	mcyesrn_x		OTHER (SPECIFY)	X		
	mcyesrn_y		DON'T KNOW	Y		
	mcyesrn_z		REFUSED	Z		
CONT		DO NOT READ: CONTINUE TO THE NEXT ITEM?	YES	1	IF item = 2	**INDIVIDUAL REFUSAL OR WITHDRAWAL**
		SELECT 'NO' ONLY IF THE PARTICIPANT HAS EXPRESSED HE/SHE DOES NOT WISH TO CONTINUE ON WITH THE SURVEY.	NO	2		
MTITLE		**MODULE 6: SEXUAL ACTIVITY**				
INSTR		In this part of the interview, I will be asking questions about your sexual relationships and practices. These questions will help us have a better understanding of how they may affect your life and risk for HIV.<br><br>Let me assure you again that your answers are completely confidential and will not be shared with anyone. If there are questions that you do not want to answer, we can go to the next question.				
601	firstsxage	How old were you when you had vaginal sex for the very first time?	AGE IN YEARS	integer	IF firstsxage = 96	[NEXT MODULE]
		Vaginal sex is when a penis enters a vagina	NEVER HAD VAGINAL SEX	96		
	firstsxagedk		DON'T KNOW	-8		
			REFUSED	-9		

NO.	VARIABLE	QUESTIONS	CODING LABELS	CODING VALUES	SKIP PATTERNS	SKIP TO
602	analsxever	People have sex in different ways. Some have vaginal sex. Some have anal sex. Anal sex is when a penis enters a person's anus. Have you ever had anal sex?	YES	1		
			NO	2		
			DON'T KNOW	-8		
			REFUSED	-9		
602a	lifetimesex	People often have sex with different people over their lifetime. In total, with how many different people have you had sex in your lifetime? Please give your best guess.	NUMBER OF SEXUAL PARTNERS IN LIFETIME	integer		
	lifetimesexdk	IF NUMBER OF SEXUAL PARTNERS IS GREATER THAN 100, ENTER '100'	DON'T KNOW	-8		
			REFUSED	-9		
603	firstsexcndm	The first time you had vaginal or anal sex, was a condom used?	YES	1		
			NO	2		
			DON'T KNOW	-8		
			REFUSED	-9		
604	condomget	If you wanted a condom, would it be easy for you to get one?	YES	1	IF condomget = 1, -8, -9	part12monum
			NO	2		
			DON'T KNOW	-8		
			REFUSED	-9		
605	cmezrsn_a	Why is it not easy for you to get a condom?	CONDOMS NOT AVAILABLE/TOO FAR	A	SKIP IF condomget != 2	
	cmezrsn_b	DO NOT READ: SELECT ALL THAT APPLY.	NOT CONVENIENT	B		
	cmezrsn_c		COSTS TOO MUCH	C		
	cmezrsn_d		EMBARASSED TO GET CONDOMS	D	IF cmezrsn_x !=X	part12monum
	cmezrsn_e		DO NOT WANT OTHERS TO KNOW	E		
	cmezrsn_f		DO NOT KNOW WHERE TO GET CONDOMS	F		
	cmezrsn_x		OTHER (SPECIFY)	X		
	cmezrsn_y		DON'T KNOW	Y		
	cmezrsn_z		REFUSED	Z		
606	part12monum	People often have sex with different partners over their lifetime. In total, with how many different people have you had sex in the last 12 months?	NUMBER OF SEXUAL PARTNERS IN LAST 12 MONTHS	integer	IF part12monum = 0,-8,-9	sellsxever
		DO NOT READ: IF NONE, ENTER '0'.	DON'T KNOW	-8		
		IF NUMBER OF SEXUAL PARTNERS IS GREATER THAN 100, ENTER '100'	REFUSED	-9		
group start						

NO.	VARIABLE	QUESTIONS	CODING LABELS	CODING VALUES	SKIP PATTERNS	SKIP TO
INSTR		Now I would like to ask you some questions about the partners you have had sex with in the last 12 months. Let me assure you again that your answers are completely confidential and will not be told to anyone. I will first ask you about your most recent partner.<br>ASK ONLY ABOU THE LAST 3 PERSONS THE PARTICIPANT HAS SEX WITH.				
607	partlivew1-3	Does the person you had sex with live in this household?	YES<br>NO	1<br>2	IF partlivew = 2	partid1-3
608	partid1-3	Please select the name below from the household membership list. Please identify the person you had sex with.	LINE NO	integer		
609	Redacted	I would like to ask you for the initials of your partner so I can keep track. They do not have to be the actual initials of your partner.	INITIALS	text		
610	partrelation1-3	What is your relationship with [INITIALS]?	HUSBAND/WIFE<br>LIVE-IN PARTNER<br>PARTNER, NOT LIVING WITH RESPONDENT<br>EX-SPOUSE/PARTNER<br>FRIEND/ACQUAINTANCE<br>SEX WORKER<br>SEX WORKER CLIENT<br>STRANGER<br>OTHER (SPECIFY)<br>DON'T KNOW<br>REFUSED	1<br>2<br>3<br>4<br>5<br>6<br>7<br>8<br>96<br>-8<br>-9	IF partrelation != 96	partgend1-3
611	partgend1-3	Is [INITIALS] male or female?	MALE<br>FEMALE<br>DON'T KNOW<br>REFUSED	1<br>2<br>-8<br>-9	SKIP IF partlivew = 1	
612	partage1-3<br>partagedk1-3	How old is [INITIALS]? Please give your best guess.	AGE IN YEARS<br>DON'T KNOW<br>REFUSED	integer<br>-8<br>-9		
613	partfirstsxtime1-3<br>partfirstsxunits1-3	How long has it been since you <u>first</u> had sex with (INITIALS)?<br>DO NOT READ: IF LESS THAN ONE WEEK RECORD IN DAYS, IF LESS THAN ONE MONTH, RECORD IN WEEKS, IF LESS THAN ONE YEAR, RECORD IN MONTHS, OTHERWISE RECORD IN YEARS.	Number of DAYS/WEEKS/MONTHS/YEARS<br>DAYS<br>WEEKS<br>MONTHS<br>YEARS<br>DON'T KNOW<br>REFUSED	integer<br>1<br>2<br>3<br>4<br>-8<br>-9		

NO.	VARIABLE	QUESTIONS	CODING LABELS	CODING VALUES	SKIP PATTERNS	SKIP TO
614	partlastsxtime1-3	How long has it been since you last had sex with (INITIALS)?	Number of DAYS/WEEKS/MONTHS	integer		
	partlastsxunits1-3	DO NOT READ: IF LESS THAN ONE WEEK RECORD IN DAYS, IF LESS THAN ONE MONTH, RECORD IN WEEKS, OTHERWISE RECORD IN MONTHS.	DAYS	1		
			WEEKS	2		
			MONTHS	3		
			DON'T KNOW	-8		
			REFUSED	-9		
615	partlastcndm1-3	The last time you had sex with [INITIALS] was a condom used?	YES	1		
			NO	2		
			DON'T KNOW	-8		
			REFUSED	-9		
616	parttimes4wks1-3	In the last 4 weeks, what is the total number of times you had sex with [INITIALS]? By "times" we mean number of sex acts. For example, you could have sex 5 times with the same partner.	NUMBER OF TIMES	integer		
	parttimes4wksdk1-3	DO NOT READ: ENTER '0' IF NONE.	DON'T KNOW	-8		
			REFUSED	-9		
617	partcondfreqvag1-3	In the last 12 months, how often did you use condoms with [INITIALS] when having vaginal sex? Was it always, most of the time, sometimes, rarely or never?	ALWAYS	1		
		(Note: Due to a skip pattern error, this question was asked to very few participants.)	MOST OF THE TIME	2		
			SOMETIMES	3		
			RARELY	4		
			NEVER	5		
			NO VAGINAL SEX IN THE LAST 12 MONTHS	6		
			DON'T KNOW	-8		
			REFUSED	-9		
618	partcondfreqanal1-3	In the last 12 months, how often did you use condoms with (INITIAL) when having anal sex? Was it always, most of the time, sometimes, rarely or never?	ALWAYS	1	SKIP IF analsxever != 1	
			MOST OF THE TIME	2		
			SOMETIMES	3		
			RARELY	4		
			NEVER	5		
			NO ANAL SEX IN THE LAST 12 MONTHS	6		
			DON'T KNOW	-8		
			REFUSED	-9		
618a	partlube1-3	In the last 12 months, how often did you use lubricant with (INITIAL) when having anal sex? Was it always, most of the time, sometimes, rarely or never?	ALWAYS	1	SKIP IF analsxever != 1	

NO.	VARIABLE	QUESTIONS	CODING LABELS	CODING VALUES	SKIP PATTERNS	SKIP TO
			MOST OF THE TIME	2		
			SOMETIMES	3		
			RARELY	4		
			NEVER	5		
			NO ANAL SEX IN THE LAST 12 MONTHS	6		
			DON'T KNOW	-8		
			REFUSED	-9		
619	partlastsup1-3	Did you enter into a sexual relationship with (INITIALS) because (INITIALS) provided you with gifts, help you to pay for things, or help you in other ways?	YES	1	SKIP IF partrelation1-3 = 6, 7	
			NO	2		
			DON'T KNOW	-8		
			REFUSED	-9		
619a	partlastsup21-3	Did you enter into a sexual relationship with (INITIALS) because you expected that (INITIALS) would provide you gifts, help you to pay for things, or help you in other ways?	YES	1	SKIP IF partrelation1-3 = 6, 7	
			NO	2	IF pastlastsup21-3= 2, -8, -9	partagain1-3
			DON'T KNOW	-8		
			REFUSED	-9		
620	partlastsupwhat_a1-3	In the last 12 months, what have you received from [INITIALS]?	DID NOT RECEIVE ANYTHING	A	IF partlastsupwhat_x1-3!= X	partagain1-3
	partlastsupwhat_b1-3		MONEY	B		
	partlastsupwhat_c1-3	SELECT ALL THAT APPLY.	FOOD	C	SKIP IF partrelation1-3 = 6, 7	
	partlastsupwhat_d1-3		SCHOOL FEES	D	SKIP IF partlastsup1-3 !=1 AND partlastsup21-3 !=1	
	partlastsupwhat_e1-3		EMPLOYMENT	E		
	partlastsupwhat_f1-3		GIFTS/FAVORS	F		
	partlastsupwhat_g1-3		TRANSPORT	G		
	partlastsupwhat_h1-3		SHELTER/RENT	H		
	partlastsupwhat_i1-3		PROTECTION	I		
	partlastsupwhat_x1-3		OTHER (SPECIFY)	X		
	no one selected		DON'T KNOW	Y		
	partlastsupwhat_z1-3		REFUSED	Z		
621	partagain1-3	Do you expect to have sex with [INITIALS] again?	YES	1		

NO.	VARIABLE	QUESTIONS	CODING LABELS	CODING VALUES	SKIP PATTERNS	SKIP TO
			NO	2		
			DON'T KNOW	-8		
			REFUSED	-9		
622	partknowhiv1-3	Does [INITIALS] know your HIV status? HIV status could mean you are HIV negative or HIV positive.	YES	1		
			NO	2		
			DON'T KNOW	-8		
			REFUSED	-9		
623	parthivsat1-3	What is the HIV status of [INITIALS]?<br>DO NOT READ: READ RESPONSES ALOUD.	THINK (INITIALS) IS POSITIVE	1		
			(INTIALS) TOLD ME HE/SHE IS POSITIVE	2		
			POSITIVE, TESTED TOGETHER	3		
			THINK (INITIALS) IS NEGATIVE	4		
			(INITIALS) TOLD ME HE/SHE IS NEGATIVE	5		
			NEGATIVE, TESTED TOGETHER	6		
			DON'T KNOW STATUS	7		
			REFUSED	-9		
624		I will now ask you about the person you have had sex with previous to (INITIALS).				
group end						
625	sellsxever	Have you ever sold sex for money?	YES	1	IF sellsxever = 2, -8, -9	buysxever
			NO	2		
			DON'T KNOW	-8		
			REFUSED	-9		
626	sellsx12mo	In the last 12 months, have you sold sex for money?	YES	1	SKIP IF sellsxever != 1	
			NO	2		
			DON'T KNOW	-8		
			REFUSED	-9		
627	sellsxcndm	The last time you sold sex for money, was a condom used?	YES	1	SKIP IF sellsxever != 1	
			NO	2		
			DON'T KNOW	-8		
			REFUSED	-9		
628	buysxever	Have you ever paid money for sex?	YES	1	IF buysxever = 2, -8, -9	[NEXT MODULE]
			NO	2		
			DON'T KNOW	-8		
			REFUSED	-9		
629	buysx12mo	In the last 12-months, have you paid money for sex?	YES	1	SKIP IF buysxever != 1	
			NO	2		
			DON'T KNOW	-8		
			REFUSED	-9		
630	buysxcndm	The last time you paid money for sex, was a condom used?	YES	1	SKIP IF buysxever != 1	
			NO	2		
			DON'T KNOW	-8		

NO.	VARIABLE	QUESTIONS	CODING LABELS	CODING VALUES	SKIP PATTERNS	SKIP TO
			REFUSED	-9		
CONT		DO NOT READ: CONTINUE TO THE NEXT ITEM?	YES	1	IF item = 2	**INDIVIDUAL REFUSAL OR WITHDRAWAL**
		SELECT 'NO' ONLY IF THE PARTICIPANT HAS EXPRESSED HE/SHE DOES NOT WISH TO CONTINUE ON WITH THE SURVEY.	NO	2		
MTITLE		**MODULE 7: HIV TESTING**				
INSTR		I would now like to ask you some questions about HIV testing.				
701	hivtstever	Have you ever **tested** for HIV?	YES	1	IF hivtstever = 1	lifetimehivtest
			NO	2	IF hivtstever = -8, -9	hivelikely
			DON'T KNOW	-8		
			REFUSED	-9		
702	hivtstnors_a	Why have you never been tested for HIV?	DON'T KNOW WHERE TO TEST	A	IF hivtstnors_x != X	hivelikely
	hivtstnors_b		TEST COSTS TOO MUCH	B		
	hivtstnors_c	DO NOT READ: SELECT ALL THAT APPLY.	TRANSPORT COSTS TOO MUCH	C		
	hivtstnors_d		TOO FAR AWAY	D		
	hivtstnors_e		AFRAID OTHERS WILL KNOW ABOUT TEST RESULTS	E		
	hivtstnors_f		DON'T NEED TEST/LOW RISK	F		
	hivtstnors_g		DID NOT RECEIVE PERMISSION FROM SPOUSE/FAMILY	G		
	hivtstnors_h		AFRAID SPOUSE/PARTNER/FAMILY WILL KNOW RESULTS	H		
	hivtstnors_i		DON'T WANT TO KNOW I HAVE HIV	I		
	hivtstnors_j		CANNOT GET TREATMENT FOR HIV	J		
	hivtstnors_k		TEST KITS NOT AVAILABLE	K		
	hivtstnors_l		RELIGIOUS REASONS	L		
	hivtstnors_x		OTHER (SPECIFY)	X		
	hivtstnors_y		DON'T KNOW	Y		
	hivtstnors_z		REFUSED	Z		
703	lifetimehivtest	In your lifetime, how many times have you been tested for HIV?	NUMBER OF TIMES	integer	SKIP IF hivtstever != 1	
	lifetimehivtestdk		DON'T KNOW	-8		
			REFUSED	-9		
704	hivtestm	What month and year was your last HIV test?	MONTH	integer		
			DON'T KNOW MONTH	-8		
			REFUSED MONTH	-9		
	hivtesty		YEARS	integer		
			DON'T KNOW YEAR	-8		
			REFUSED YEAR	-9		
705	hivtstlocation	Where was the last test done?	VCT FACILITY	1	IF hivtstlocation != 96	hivtstrslt

NO.	VARIABLE	QUESTIONS	CODING LABELS	CODING VALUES	SKIP PATTERNS	SKIP TO
			MOBILE VCT	2		
			AT HOME	3		
			HEALTH CLINIC / FACIITY	4		
			HOSPITAL OUTPATIENT CLINIC	5		
			TB CLINIC	6		
			STI CLINIC	7		
			HOSPITAL INPATIENT WARDS	8		
			BLOOD DONATING CENTER	9		
			ANC CLINIC	10		
			OTHER (SPECIFY)	96		
			DON'T KNOW	-8		
			REFUSED	-9		
706	hivtstrslt	What was the result of that HIV test?	POSITIVE	1	IF hivtstrslt = 2, 3, 4, -8, -9	hivlikely
			NEGATIVE	2	IF hivtstrslt = 1	hivtm/hivty
			UNCERTAIN/INDETERMINATE	3		
			DID NOT RECEIVE THE RESULT	4		
			DON'T KNOW	-8		
			REFUSED	-9		
707	hivlikely	How likely do you think it is that you will get HIV?	VERY LIKELY	1		
			SOMEWHAT LIKELY	2		
			NOT LIKELY	3		
			DON'T KNOW	-8		
			REFUSED	-9		
INSTR		Please tell me if you strongly agree, agree, disagree or strongly disagree with the following statements [ASKED OF ALL PARTICIPANTS]				
708	allowwork	A person with HIV/AIDS should be allowed to work with other people	STRONGLY AGREE	1		
			AGREE	2		
			DISAGREE	3		
			STRONGLY DISAGREE	4		
			DON'T KNOW	-8		
			REFUSED	-9		
709	compassion	People who have HIV/AIDS deserve compassion	STRONGLY AGREE	1		
			AGREE	2		
			DISAGREE	3		
			STRONGLY DISAGREE	4		
			DON'T KNOW	-8		
			REFUSED	-9		
710	loserespect	People who are suspected of having HIV/AIDS lose respect in the community	STRONGLY AGREE	1		
			AGREE	2		
			DISAGREE	3		
			STRONGLY DISAGREE	4		
			DON'T KNOW	-8		
			REFUSED	-9		

NO.	VARIABLE	QUESTIONS	CODING LABELS	CODING VALUES	SKIP PATTERNS	SKIP TO
711	evtest	Everyone should get tested for HIV.	STRONGLY AGREE	1		
			AGREE	2		
			DISAGREE	3		
			STRONGLY DISAGREE	4		
			DON'T KNOW	-8		
			REFUSED	-9		
712	hivonly	Only persons who think they might be infected with HIV should get an HIV test.	STRONGLY AGREE	1		
			AGREE	2		
			DISAGREE	3		
			STRONGLY DISAGREE	4		
			DON'T KNOW	-8		
			REFUSED	-9		
713	startimm	I would be willing to start treatment immediately if this would make me less infectious to a sexual partner, even if there was no benefit to my own health.	STRONGLY AGREE	1		
			AGREE	2		
			DISAGREE	3		
			STRONGLY DISAGREE	4		
			DON'T KNOW	-8		
			REFUSED	-9		
714	startsck	I would not want to start treatment now when I am feeling healthy because only people who are very sick need to take ARVs	STRONGLY AGREE	1		
			AGREE	2		
			DISAGREE	3		
			STRONGLY DISAGREE	4		
			DON'T KNOW	-8		
			REFUSED	-9		
715	hivtfposm	What was the month and year of your first HIV positive test result? Please give your best guess.	MONTH	date	SKIP IF lifetimehivtest = 1	
		This will be the very first HIV positive test result that you have received	DON'T KNOW MONTH	-8	SKIP IF hivtstever !=1	
		DO NOT READ: PROBE TO VERIFY DATE.	REFUSED MONTH	-9		
	hivtfposy		YEAR	date		
			DON'T KNOW YEAR	-8		
			REFUSED YEAR	-9		
716	hivpostell_a	Who have you told that you are HIV positive?	NO ONE	A	IF hivpostold != X	next item
	hivpostell_b	DO NOT READ: CHECK ALL THAT APPLY.	SPOUSE/SEX PARTNER	B		
	hivpostell_c		DOCTOR	C	SKIP IF hivtstrslt! =1	
	hivpostell_d		FRIEND	D		
	hivpostell_e		FAMILY MEMBER	E		
	hivpostell_x		OTHER (SPECIFY)	X		
	hivpostell_y		DON'T KNOW	Y		
	no one selected		REFUSED	Z		

NO.	VARIABLE	QUESTIONS	CODING LABELS	CODING VALUES	SKIP PATTERNS	SKIP TO
INSTR		Now I would like to ask you questions about your experiences with health care providers.			SKIP IF hivtstrslt! =1	
717	hivstathide	In the last 12 months, when you sought health care in a facility where your HIV status is not known, did you feel you needed to hide your HIV status?	YES	1	SKIP IF hivtstrslt! =1	
			NO, NO NEED TO HIDE	2		
			NO, DID NOT ATTEND HEALTH FACILITY IN LAST 12 MONTHS	3		
			DON'T KNOW	-8		
			REFUSED	-9		
718	hivstatdeniedcare	In the last 12 months, have you been denied health services including dental care, because of your HIV status?	YES	1	SKIP IF hivtstrslt! =1	
			NO	2		
			NO ONE KNOWS MY STATUS	3		
			DON'T KNOW	-8		
			REFUSED	-9		
CONT		DO NOT READ: CONTINUE TO THE NEXT ITEM?	YES	1	IF item=2	**INDIVIDUAL REFUSAL OR WITHDRAWAL**
		SELECT 'NO' ONLY IF THE PARTICIPANT HAS EXPRESSED HE/SHE DOES NOT WISH TO CONTINUE ON WITH THE SURVEY.	NO	2		
MTITLE		**MODULE 8: HIV STATUS, CARE AND TREATMENT**				
INSTR		Now I'm going to ask you more about your experience with HIV support, care and treatment.			SKIP MODULE IF hivtstrslt = 2, 3, 4, -8, -9	
801	hivcare	After learning you had HIV, have you ever received HIV medical care and treatment from a doctor, clinical officer or nurse?	YES	1	IF hivcare = 1	hivcfm/hivcfy
			NO	2	IF hivcare = -8, -9	cd4testever
			DON'T KNOW	-8		
			REFUSED	-9		
802	hivcnotrsn	What is the main reason why you have never received HIV medical care and treatment from a doctor, clinical officer or nurse?	FACILITY IS TOO FAR AWAY	1	IF hivcnotrsn != 96	cd4testever
			I DON'T KNOW WHERE TO GET HIV MEDICAL CARE	2		
			COST OF CARE	3		
			COST OF TRANSPORT	4		
			I DO NOT NEED IT/I FEEL HEALTHY/NOT SICK	5		
			I FEAR PEOPLE WILL KNOW THAT I HAVE HIV IF I GO TO A CLINIC	6		
			RELIGIOUS REASONS	7		
			I'M TAKING TRADITIONAL MEDICINE	8		

NO.	VARIABLE	QUESTIONS	CODING LABELS	CODING VALUES	SKIP PATTERNS	SKIP TO
			DO NOT TRUST THE STAFF/QUALITY OF CARE	9		
			OTHER (SPECIFY)	96		
			DON'T KNOW	-8		
			REFUSED	-9		
803	hivcfm	What month and year did you first see a doctor, clinical officer or nurse for HIV medical care and treatment?	MONTH	date		
		DO NOT READ: PROBE TO VERIFY DATE.	DON'T KNOW MONTH	-8		
			REFUSED MONTH	-9		
	hivcfy		YEAR	date		
			DON'T KNOW YEAR	-8		
			REFUSED YEAR	-9		
804	hivclm	What month and year did you last see a doctor, clinical officer or nurse for HIV medical care?	MONTH	date	IF [CURRENT DATE] - hivclm/hivcly <= 7 MONTHS OR hivcml/hivcly = -8, -9	hivcdistance
			DON'T KNOW MONTH	-8		
			REFUSED MONTH	-9		
	hivcly		YEAR	date		
			DON'T KNOW YEAR	-8		
			REFUSED	-9		
805	hivcnot6mo	What is the main reason for not seeing a doctor, clinical officer or nurse for HIV medical care in the past 6 months?	FACILITY IS TOO FAR AWAY	1	IF hivcnot6mo != 96	cd4testever
			I DON'T KNOW WHERE TO GET HIV MEDICAL CARE	2		
			COST OF CARE	3		
			COST OF TRANSPORT	4		
			I DO NOT NEED IT/I FEEL HEALTHY/NOT SICK	5		
			I FEAR PEOPLE WILL KNOW THAT I HAVE HIV IF I GO TO A CLINIC	6		
			RELIGIOUS REASONS	7		
			I'M TAKING TRADITIONAL MEDICINE	8		
			NO APPOINTMENT SCHEDULED/DID NOT MISS MOST RECENT APPOINTMENT	9		
			OTHER (SPECIFY)	96		
			DON'T KNOW	-8		
			REFUSED	-9		
806	cd4testever	Have you ever had a CD4 count test?	YES	1	IF cd4testever = 2, -8, -9 AND HIVCARE = 1	arvstakenev
		The CD4 count tells you how sick you are with HIV and if you need to take ARVs or other HIV medications.	NO	2	IF cd4testever = 2, -8, -9 AND HIVCARE = 2, -8, -9	[NEXT MODULE]
			DON'T KNOW	-8		
			REFUSED	-9		

NO.	VARIABLE	QUESTIONS	CODING LABELS	CODING VALUES	SKIP PATTERNS	SKIP TO
807	cd4ttm	What month and year were you last tested for your CD4 count?	MONTH	date		
			DON'T KNOW MONTH	-8		
			REFUSED MONTH	-9		
	cd4tty		YEAR	date		
			DON'T KNOW YEAR	-8		
			REFUSED YEAR	-9		
808	arvstakenev	Have you <u>ever</u> taken ARVs, that is, antiretroviral medications to treat HIV infection?	YES	1	IF arvstakenev = 1	arvftm/arvfty
			NO	2	IF arvstakenev = -8, -9	[NEXT MODULE]
			DON'T KNOW	-8		
			REFUSED	-9		
809	arvsnottake	What is the main reason you have never taken ARVs?	NOT ELIGIBLE FOR TREATMENT	1	IF arvsnottake != 96	[NEXT MODULE]
			HEALTH CARE PROVIDER DID NOT PRESCRIBE	2		
			HIV MEDICINES NOT AVAILABLE	3		
			I FEEL HEALTHY/NOT SICK	4		
			COST OF MEDICATIONS	5		
			COST OF TRANSPORT	6		
			RELIGIOUS REASONS	7		
			TAKING TRADITIONAL MEDICATIONS	8		
			NOT ATTENDING HIV CLINIC	9		
			OTHER (SPECIFY)	96		
			DON'T KNOW	-8		
			REFUSED	-9		
810	arvftm	What month and year did you <u>first</u> start taking ARVs?<br>DO NOT READ: PROBE TO VERIFY DATE.	MONTH	date		
			DON'T KNOW MONTH	-8		
			REFUSED MONTH	-9		
	arvfty		YEAR	date		
			DON'T KNOW YEAR	-8		
			REFUSED YEAR	-9		
811	arvscurrent	Are you <u>currently</u> taking ARVs, that is, antiretroviral medications?	YES	1	IF arvscurrent = 1	arvsmissdays
		By currently, I mean that you may have missed some doses but you are still taking ARVs.	NO	2	IF arvscurrent = -8, -9	[NEXT MODULE]
			DON'T KNOW	-8		
			REFUSED	-9		
812	arvsnotcurrsn	Can you tell me the <u>main</u> reason why you are <u>not</u> currently taking ARVs?	I HAVE TROUBLE TAKING A TABLET EVERYDAY	1	IF arvsnotcurrsn != 96	[NEXT MODULE]
			I HAD SIDE EFFECTS	2		
			FACILITY TOO FAR AWAY FOR ME TO GET MEDICINE REGULARLY	3		
			COST OF MEDICATIONS	4		
			COST OF TRANSPORT	5		
			I FEEL HEALTHY/NOT SICK	6		

NO.	VARIABLE	QUESTIONS	CODING LABELS	CODING VALUES	SKIP PATTERNS	SKIP TO
			FACILITY WAS OUT OF STOCK	7		
			RELIGIOUS REASONS	8		
			TAKING TRADITIONAL MEDICATIONS	9		
			OTHER (SPECIFY)	96		
			DON' T KNOW	-8		
			REFUSED	-9		
816	arvsmissdays	People sometimes forget to take all of their ARVs every day. In the past 30 days, how many days have you missed taking any of your ARV pills ?	NUMBER OF DAYS	integer		
	arvsmissdaysdk	DO NOT READ: CODE '0' IF NONE.	DON'T KNOW	-8		
			REFUSED	-9		
817	hivcdistance	At your last HIV care visit, approximately how long did it take you to travel from your home (or workplace) one way?	LESS THAN ONE HOUR	1		
			ONE TO TWO HOURS	2		
			MORE THAN TWO HOURS	3		
			DON'T KNOW	-8		
			REFUSED	-9		
818	hivccost	At your last HIV care visit, approximately how much did it cost to travel from your home (or workplace) one way?	COST	integer		
	hivccostdk	ENTER COST IN EMALENGENI.	DON'T KNOW	-8		
			REFUSED	-9		
CONT		DO NOT READ: CONTINUE TO THE NEXT ITEM?	YES	1	IF item=2	**INDIVIDUAL REFUSAL OR WITHDRAWAL**
		SELECT 'NO' ONLY IF THE PARTICIPANT HAS EXPRESSED HE/SHE DOES NOT WISH TO CONTINUE ON WITH THE SURVEY.	NO	2		
MTITLE		**MODULE 9: TUBERCULOSIS AND OTHER HEALTH ISSUES**				
INSTR		Now I will ask you about tuberculosis or TB.				
901	tbclinvisit	Have you ever visited a TB clinic (or health facility) for TB diagnosis or treatment?	YES	1	IF tbclinvisit = 2, -8, -9	[NEXT MODULE]
			NO	2		
			DON'T KNOW	-8		
			REFUSED	-9		
902	tbdiagn	Have you ever been told by a doctor, clinical officer or nurse that you had TB?	YES	1	IF tbdiagn = 2, -8, -9	[NEXT MODULE]
			NO	2		
			DON'T KNOW	-8		
			REFUSED	-9		
903	tbtreated	Were you ever treated for TB?	YES	1	IF tbtreated = 2, -8, -9	[NEXT MODULE]
			NO	2		
			DON'T KNOW	-8		
			REFUSED	-9		
904	tbttretcurr	Are you currently on treatment for TB?	YES	1		
			NO	2		

NO.	VARIABLE	QUESTIONS	CODING LABELS	CODING VALUES	SKIP PATTERNS	SKIP TO
			DON'T KNOW	-8		
			REFUSED	-9		
905	tbtreat6mofull	The last time you were treated for TB, did you complete at least 6 months of treatment?	YES	1		
			NO, MEDICINE WAS STOPPED IN LESS THAN 6 MONTHS	2		
			NO, BUT I AM STILL ON TREATMENT	3		
			DON'T KNOW	-8		
			REFUSED	-9		
CONT		DO NOT READ: CONTINUE TO THE NEXT ITEM?	YES	1	IF item = 2	**INDIVIDUAL REFUSAL OR WITHDRAWAL**
		SELECT 'NO' ONLY IF THE PARTICIPANT HAS EXPRESSED HE/SHE DOES NOT WISH TO CONTINUE ON WITH THE SURVEY.	NO	2		
MCTITLE		**OPTIONAL MODULE C: ALCOHOL USE**				
INSTR		The next few questions will be on your use of alcohol.<br>Remember, all the answers you provide will be kept confidential.				
C1	alcfreq	How often do you have a drink containing alcohol?<br>DO NOT READ: SHOW ALCOHOL AID.	NEVER	0	IF alcfreq = 0, -8, -9	[NEXT MODULE]
			MONTHLY OR LESS	1		
			2-4 TIMES A MONTH	2		
			2-3 TIMES A WEEK	3		
			4 OR MORE TIMES A WEEK	4		
			DON'T KNOW	-8		
			REFUSED	-9		
C2	alcnumday	How many drinks containing alcohol do you have on a typical day?	NONE OR LESS THAN 1	0	SKIP IF alcfreq = 0, -8, -9	
			1 OR 2	1		
			3 OR 4	2		
			5 OR 6	3		
			7 TO 9	4		
			10 OR MORE	5		
			DON'T KNOW	-8		
			REFUSED	-9		
C3	alcsixmore	How often do you have six or more drinks on one occasion?	NEVER	0	SKIP IF alcfreq = 0, -8, -9	
			LESS THAN MONTHLY	1		
			MONTHLY	2		
			WEEKLY	3		
			DAILY OR ALMOST DAILY	4		
			DON'T KNOW	-8		
			REFUSED	-9		
CONT		DO NOT READ: CONTINUE TO THE NEXT ITEM?	YES	1	IF item = 2	**INDIVIDUAL REFUSAL OR WITHDRAWAL**

NO.	VARIABLE	QUESTIONS	CODING LABELS	CODING VALUES	SKIP PATTERNS	SKIP TO
		SELECT 'NO' ONLY IF THE PARTICIPANT HAS EXPRESSED HE/SHE DOES NOT WISH TO CONTINUE ON WITH THE SURVEY.	NO	2		
MTITLE		**MODULE 10: GENDER NORMS**			SKIP IF curmar = 3, 4, 5, -8, -9	
INSTR		Now I would like to ask you question on attitudes and decision-making in your home.				
1001	healthc	Who usually makes decisions about health care for yourself: you, your (spouse/partner), you and your (spouse/partner) together, or someone else?	I DO	1		
			SPOUSE/PARTNER	2		
			WE BOTH DO	3		
			SOMEONE ELSE	4		
			DON'T KNOW	-8		
			REFUSED	-9		
1002	money	Who generally decides about how the money you receive is spent: you, your (spouse/partner), you and your (spouse/partner) together, or someone else?	I DO	1		
			SPOUSE/PARTNER	2		
			WE BOTH DO	3		
			SOMEONE ELSE	4		
			DON'T KNOW	-8		
			REFUSED	-9		
CONT		DO NOT READ: CONTINUE TO THE NEXT ITEM?	YES	1	IF item = 2	**INDIVIDUAL REFUSAL OR WITHDRAWAL**
		SELECT 'NO' ONLY IF THE PARTICIPANT HAS EXPRESSED HE/SHE DOES NOT WISH TO CONTINUE ON WITH THE SURVEY.	NO	2		
MTITLE		MODULE 11: VIOLENCE			IF gender = 1 OR IF gender = 2 AND NOT SELECTED	REFER
INSTR11		You have been selected to be asked questions on other important aspects of a person's life. I know that some of these questions are very personal. However, your answers are important for helping to understand the condition of men and women in Swaziland. Let me assure you that your answers are completely confidential and will not be told to anyone and no one in your household will know that you were asked these questions.<br><br>By sex, we mean vaginal, anal, oral sex or the insertion of an object into your vagina or anus. Vaginal sex is when a penis enters a vagina. Anal sex is when a penis enters an anus (butt). Oral sex is when a partner puts his/her mouth on his/her partner's penis or vagina.				
SUBTITLE		**SEXUAL VIOLENCE**				
D0	touchever	Have you ever been touched by someone in a sexual way without your permission?	YES	1	IF touchever = 2, -8, -9	frcsxtimes

NO.	VARIABLE	QUESTIONS	CODING LABELS	CODING VALUES	SKIP PATTERNS	SKIP TO
		Touching in a sexual way without permission includes fondling, pinching, grabbing, or touching you on or around your sexual body parts.	NO	2		
			DON'T KNOW	-8		
			REFUSED	-9		
D1	touchtimes	How many times has anyone ever touched you in a sexual way without your permission, but did not try and force you to have sex?	NUMBER OF TIMES	integer	IF touchtimes = -8, -9	frcsxtimes
	touchtimesdk	Touching in a sexual way without permission includes fondling, pinching, grabbing, or touching you on or around your sexual body parts.	DON'T KNOW	-8		
			REFUSED	-9		
D2	touchage	How old were you the first time this happened?	AGE IN YEARS	integer		
	touchagedk		DON'T KNOW	-8		
			REFUSED	-9		
D15	frcsxtimes	How many times in your life have you been physically forced to have sex?	NUMBER OF TIMES	integer	IF frcsxtimes = 0, -8, -9	unwntseek
	frcsxtimesdk	CODE '0' IF NONE.	DON'T KNOW	-8		
			REFUSED	-9		
D16	frcsxage	How old were you the first time someone physically forced you to have sex?	AGE IN YEARS	integer		
	frcsxagedk		DON'T KNOW	-8		
			REFUSED	-9		
D19	frcsx12mo	In the past 12 months, did someone physically force you to have sex?	YES	1	IF frcsx12mo = 2, -8, -9	unwntseek
			NO	2		
			DON'T KNOW	-8		
			REFUSED	-9		
D20	frcsx12mowho	In the last 12 months, who physically forced you to have sex?	PARTNER	A	IF frcsx12mowho !=X	unwntseek
			RELATIVE	B		
		By partner, I mean a sexual partner whether or not you were married at the time.	STRANGER	C		
			OTHER	X		
			DON'T KNOW	Y		
			REFUSED	Z		
D31	uwntsxhelp_a	After any of these unwanted sexual experiences, did you try to seek professional help or services from any of the following?	I DID NOT TRY TO SEEK HELP	A	SKIP IF (touchtimes = 0, -8, -9) AND (frcsxtimes = 0, -8, -9)	
	uwntsxhelp_b	DO NOT READ: READ RESPONSES ALOUD.	HEALTHCARE PROFESSIONAL	B	IF unwntseek = A	unwntsxnohlp
	uwntsxhelp_c	SELECT ALL THAT APPLY.	POLICE OR OTHER SECURITY PERSONNEL	C	IF unwntseek = B, C, D, E, Y, Z	vlnc12motimes
	uwntsxhelp_d		SOCIAL WORKER, COUNSELOR OR NON-GOVERNMENTAL ORGANIZATION	D	IF unwntseek = X	unwntseekoth
	uwntsxhelp_e		RELIGIOUS LEADER	E		

NO.	VARIABLE	QUESTIONS	CODING LABELS	CODING VALUES	SKIP PATTERNS	SKIP TO
	uwntsxhelp_x		OTHER (SPECIFY)	X		
	uwntsxhelp_y		DON'T KNOW	Y		
	uwntsxhelp_z		REFUSED	Z		
D32	unwntsxnohlp	What was the main reason that you did not try to seek help or services?	DID NOT KNOW SERVICES WERE AVAILABLE	1	IF unwntsxnohlp != 96	vlnc12motimes
			SERVICES NOT AVAILABLE	2		
			AFRAID OF GETTING IN TROUBLE	3		
			ASHAMED FOR SELF/FAMILY	4		
			COULD NOT AFFORD SERVICES	5		
			DID NOT THINK IT WAS A PROBLEM	6		
			FELT IT WAS MY FAULT	7		
			AFRAID OF BEING ABANDONED	8		
			DID NOT NEED/WANT SERVICES	9		
			AFRAID OF MAKING SITUATION WORSE	10		
			OTHER (SPECIFY)	96		
			DON'T KNOW	-8		
			REFUSED	-9		
SUBTITLE		**PHYSICAL VIOLENCE**				
D35	vlnc12motimes	In the past 12 months, how many times did someone:	NOT IN LAST 12 MONTHS	1	IF vlnc12motimes = 1, -8, -9	END
		- Punched, kicked, whipped, or beat you with an object	ONCE	2		
		- Slapped you, threw something at you that could hurt you, pushed you or shoved you	FEW	3		
		- Choked, smothered, tried to drown you, or burned you intentionally	MANY	4		
		- Used or threatened you with a knife, gun or other weapon?	DON'T KNOW	-8		
			REFUSED	-9		
D35a	vlnc12mowho	In the last 12 months, who did any of these things to you?	PARTNER	A	IF vlnc12mowho ! = X	seekhelp
			RELATIVE	B		
		By partner, I mean a sexual partner whether or not you were married at the time.	STRANGER	C		
			OTHER	X		
			DON'T KNOW	Y		
			REFUSED	Z		
D41	seekhelp_a	Thinking about all these experiences that we just discussed, whether someone has done the following:	I DID NOT TRY TO SEEK HELP	A	IF seekhelp = A	seekhelpwhynot
	seekhelp_b	- Punched, kicked, whipped, or beat you with an object	HEALTHCARE PROFESSIONAL	B	IF seekhelp = B, C, D, E, Y, Z	END
	seekhelp_c	- Slapped you, threw something at you that could hurt you, pushed you or shoved you	POLICE OR OTHER SECURITY PERSONNEL	C		
	seekhelp_d	- Choked, smothered, tried to drown you, or burned you intentionally	SOCIAL WORKER, COUNSELOR OR NON-GOVERNMENTAL ORGANIZATION	D		

NO.	VARIABLE	QUESTIONS	CODING LABELS	CODING VALUES	SKIP PATTERNS	SKIP TO
	seekhelp_e	- Used or threatened you with a knife, gun or other weapon	RELIGIOUS LEADER	E		
	seekhelp_x	Did you try to seek professional help or services for any of these incidents from any of the following?	OTHER (SPECIFY)	X		
	seekhelp_y	DO NOT READ: READ RESPONSES ALOUD.	DON'T KNOW	Y		
	seekhelp_z	SELECT ALL THAT APPLY.	REFUSED	Z		
D42	seekhelpwhynot	What was the main reason that you did not try to seek help or services?	DID NOT KNOW SERVICES WERE AVAILABLE	1	IF seekhelpwhynot != 96	END
			SERVICES NOT AVAILABLE	2		
			AFRAID OF GETTING IN TROUBLE	3		
			ASHAMED FOR SELF/FAMILY	4		
			COULD NOT AFFORD SERVICES	5		
			DID NOT THINK IT WAS A PROBLEM	6		
			FELT IT WAS MY FAULT	7		
			AFRAID OF BEING ABANDONED	8		
			DID NOT NEED/WANT SERVICES	9		
			AFRAID OF MAKING SITUATION WORSE	10		
			OTHER (SPECIFY)	96		
			DON'T KNOW	-8		
			REFUSED	-9		
INSTR		Thank you for taking the time to participate in this survey. Your responses will be very helpful to the Ministry of Health to better understand how to improve health programs in the country.<br><br>DO NOT READ: PROVIDE PARTICIPANT WITH LIST OF ORGANIZATIONS, IF NOT ALREADY GIVEN.				
COMMENT		DO NOT READ: COMMENTS FROM INTERVIEWER		text	IF INCONBIO = 1 OR INFUTCON = 1 OR ASBIO15P = 1 OR ASFUTCON = 1	**LOCATOR**
					IF (INCONBIO = 2 AND INFUTCON = 2) OR (ASBIO15P = 2 AND ASFUTCON = 2)	**BIOMARKER REFUSAL**
