# Supplementary material for: HIV incidence, viremia, and the national response in Eswatini: Two sequential population-based surveys
Source: PLoS One. 2021 Dec 2;16(12):e0260892. doi: 10.1371/journal.pone.0260892 (PMC8639055; doi:10.1371/journal.pone.0260892)
Supplement: S5 File — (DOCX) [file pone.0260892.s005.docx]

U**Table of Contents**

Investigators 2

Supporting methods 3

Supporting tables 5

References 11

U**Investigators**

Rejoice NkambuleP^1*^P, Neena M. PhilipP^2*^P, Giles ReidP^2^P, Zandile MnisiP^1^P, Harriet Nuwagaba-BiribonwohaP^3,4^P, Tony T. AoP^5^P, Choice GinindzaP^6^P, Yen T. DuongP^2^P, Hetal PatelP^7^P, Suzue SaitoP^2^P, Chelsea SolmoP^2^P, Kristin BrownP^7^P, Chiara S. MooreP^8^P, Andrew C. VoetschP^7^P, George BicegoP^7^P, Naomi BockP^7^P, Fortune MhlangaP^6^P, Tengetile DlaminiP^9^P, Khanya MabuzaP^9^P, Amos ZwaneP^6^P, Ruben SahaboP^4^P, Trudy DobbsP^7^P, Bharat ParekhP^7^P, Wafaa El-SadrP^2,3^P, Caroline RyanP^5^P, Jessica JustmanP^2,3^P

U**Affiliations**

P^1^PMinistry of Health, Mbabane, Eswatini

P^2^PICAP at Columbia University, Mailman School of Public Health, New York, United States

P^3^PDepartment of Epidemiology, Columbia University, Mailman School of Public Health, New York, United States

P^4^PICAP at Columbia University, Mailman School of Public Health, Mbabane, Eswatini

P^5^PU.S. Centers for Disease Control and Prevention Eswatini, Mbabane, Eswatini

P^6^PCentral Statistics Office, Mbabane, Eswatini

P^7^PU.S. Centers for Disease Control and Prevention, Center for Global Health, Division of Global HIV and TB, Atlanta, United States

P^8^PPHI/CDC Global Health Fellowship Program, Oakland, United States

P^9^PNational Emergency Response Council on HIV/AIDS (NERCHA), Mbabane, Eswatini

* co-lead authorship

U**Supporting methods**

**Data sources, variables, and laboratory assessments**

Study teams administered either paper-based (SHIMS1) or tablet-based (SHIMS2) face-to-face interviews in a private location in or near the home using siSwati or English questionnaires on demographic, clinical, and behavioural measures. Data included self-reported status of male circumcision, prior HIV diagnosis, antiretroviral (ART) uptake, and age, using identical or very similar questions in both surveys. Characteristics of the three most recent sexual partners, including condom use, referred to recall periods of six months in SHIMS1 and 12 months in SHIMS2. Response options on frequency of condom use were re-categorized to strengthen comparability.

A study nurse and/or counsellor provided HIV counselling with condoms and collected whole blood using venipuncture or, in select cases in SHIMS2, finger prick. Field-based HIV rapid testing was conducted with return of results during the household visit and referral to HIV care per national guidelines for those testing HIV-seropositive. All samples were transported on a daily basis to the national reference laboratory for processing into plasma (or dried blood spot (DBS) specimens among finger prick samples in SHIMS2), quality assurance testing, and 80°C freezer storage. Quality assurance and control procedures were done throughout the testing periods for all biomarker assays.

HIV prevalence estimates in both surveys are based on the number of individuals testing HIV-seropositive. Rapid HIV testing was initiated with either Determine HIV-1/2Ag/Ab Combo (SHIMS1) or Determine HIV-1/2 (SHIMS2) (Alere Determine™ HIV-1/2 (Abbot Laboratories, Lake Bluff, IL)). Determine-reactive samples were confirmed with Uni-Gold™HIV Test (Trinity Biotech, Wicklow, Ireland). Indeterminate results in SHIMS1 were resolved using a two-test HIV EIA algorithm (Genscreen HIV-1/2 Version 2 Assay, Bio-Rad Laboratories, Hercules, California, USA; and Vironostika HIV Uni-Form II plus O, bioMerieux Inc., Marcy-l'Étoile, France). In SHIMS2, discrepant results were resolved using Clearview Complete HIV-1/2 (Abbot Laboratories, Lake Bluff, IL)) and confirmed with Geenius HIV 145T/2 Confirmatory Assay (Bio-Rad, Hercules, California, USA)45T. The final HIV status in both surveys was returned to participants. In SHIMS1, all HIV-seronegative samples underwent nucleic acid amplification testing (NAAT) using pools of ten samples to identify acute HIV infection [1, 2].P Individuals with NAAT-positive results received follow-up visits within six months to confirm seroconversion.

All seropositive plasma samples were tested for viral load at the national laboratory using the COBAS AmpliPrep/TaqMan HIV-1 Test, v2.0 automated platform (Roche, USA), according to manufacturers’ instructions. For the 46 (1.5%) seropositive participants without available plasma specimens in SHIMS2, dried blood spot samples were instead tested using a modified Abbott assay for elution. HIV viral load suppression estimates in both surveys are based on the number of HIV-seropositive individuals with a HIV viral load of <1000 copies/mL. Unsuppressed HIV viremia estimates are based on the number of HIV-seropositive individuals with a HIV viral load ≥1,000 copies/mL among all persons, regardless of HIV serostatus.

HIV incidence estimates in both surveys are based on the number of HIV-seropositive individuals testing with a recent infection. All HIV-seropositive samples were tested for recent HIV infection using HIV-1 Limiting Antigen (LAg) Avidity Assay. Testing procedures are described elsewhere [3, 4]. Specimens with a normalized optical density (ODn) value ≤2.0 were confirmed by further testing of the sample in triplicate, using the median value as the final ODn result for that specimen. Specimens with ODn values ≤1.5 and viral load ≥ 1000 copies/mL were classified as recent infections. Specimens with ODn values ≤1.5 and viral load < 1000 copies/mL were re-classified as long-term infections.

**Statistical methods**

Design weights comprised the selection probabilities of enumeration areas and of households. In SHIMS1, non-response adjustments were based on a four-way cross-tabulation of age, gender, rural/urban, and region, as collected in the household enumeration. In SHIMS2, non-response adjustment used the Least Absolute Shrinkage and Selection Operator (LASSO)[5] procedure to identify input variables for a chi-square automatic interaction detection (CHAID)[6] model. Post-stratification adjustments were based on the 2007 Swaziland Census [7] counts by age, gender, rural/urban, and region in SHIMS1 and on the 2017 Swaziland national projections [8] by gender and five-year age groups in SHIMS2.

Recency data was missing for 66 (1%) of HIV-positive participants in SHIMS1 and 1 (0.04%) HIV-positive participant in SHIMS2. A total of 0.2% of HIV-positive participants in SHIMS1 and SHIMS2 were excluded from HIV continuum of care estimations due to missing data on awareness of HIV status, current ART use, and/or viral load. Viral load data was missing among 44 (0.8%) and 0 HIV-positive participants in SHIMS1 and SHIMS2, respectively.

Regarding participant characteristics, demographic data in SHIMS1 was missing among 4% or less of participants. Approximately 10% of data on number of sexual partners was also missing. In SHIMS2, more than 1 percent of data was missing on only two measures: number of sexual partners (1.3%) and frequency of condom use (30%).

U**Supporting tables**

**S1 Table: Participation among adults 18-49 years in Eswatini, 2011 and 2016 surveys.**

|  |  | **2011** | | | **2016** | | |  |
| --- | --- | --- | --- | --- | --- | --- | --- | --- |
|  |  | **Urban** | **Rural** | **Total** | **Urban** | **Rural** | **Total** |  |
| **Households** | |  |  |  |  |  |  |  |
|  | Selected (#) | 4,208 | 10,683 | 14,891 | 1,863 | 4,554 | 6,417 |  |
|  | Occupied (#) | 3,736 | 9,599 | 13,335 | 1,757 | 4,299 | 6,056 |  |
|  | Interviewed (#) | 3,523 | 9,048 | 12,571 | 1,408 | 3,777 | 5,185 |  |
|  | Response (%)^*^ | 94 | 94 | 94 | 80 | 88 | 86 |  |
| **Women, 18-49 years** | |  |  |  |  |  |  |  |
|  | Eligible (#) | 3,762 | 9,874 | 13,636 | 1,194 | 3,640 | 4,834 |  |
|  | Completed interview and blood draw (#) | 3,195 | 7,847 | 11,042 | 1,005 | 3,247 | 4,252 |  |
|  | Individual response rate^†^ (%) | 85 | 79 | 81 | 84 | 89 | 88 |  |
|  | Weighted individual response rate (%) | 85 | 80 | 81 | 85 | 89 | 88 |  |
|  | Composite response (%)^‡^ | 80 | 75 | 76 | 67 | 78 | 75 |  |
|  | Weighted composite response (%) | 80 | 75 | 76 | 68 | 78 | 75 |  |
| **Men, 18-49 years** | |  |  |  |  |  |  |  |
|  | Eligible (#) | 3,037 | 8,045 | 11,082 | 994 | 2,905 | 3,899 |  |
|  | Completed interview and blood draw (#) | 2,066 | 5,064 | 7,130 | 761 | 2,252 | 3,013 |  |
|  | Individual response rate^†^ (%) | 68 | 63 | 64 | 77 | 78 | 77 |  |
|  | Weighted individual response rate (%) | 67 | 63 | 64 | 76 | 77 | 77 |  |
|  | Composite response (%)^‡^ | 64 | 59 | 61 | 61 | 68 | 66 |  |
|  | Weighted composite response (%) | 63 | 60 | 60 | 61 | 68 | 66 |  |
| **Total individuals with blood drawn (#)** | | 5,261 | 5,261 | 12,911 | 18,172 | 1,766 | 5,499 |  |
|  | * Defined as the proportion of interviewed households among occupied households. | | | | | | | |
|  | † Defined as the proportion of eligible people within participating households who completed an interview and blood draw.  ‡ Defined as the product of household and individual response rates. | | | | | | | |

**S2 Table: HIV prevalence, incidence, viral load suppression, and unsuppressed viremia by sex and age group in Eswatini, 2011 and 2016.**

|  | **2011** | | **2016** | | **2011 versus 2016** | |  |
| --- | --- | --- | --- | --- | --- | --- | --- |
|  | **n/N** | **% (95% CI)** | **n/N** | **% (95% CI)** | **Risk ratio (95% CI)** | **p-value** | |
| **HIV prevalence** | | | | | | | |
| **18-24 years** | 1170/6962 | 16 (15 - 17) | 276/2333 | 11 (10 - 12) | 0.67 (0.58 - 0.78) | <.001 | |
| **Women** | 1039/4007 | 27 (25 - 28) | 233/1296 | 18 (15 - 20) | 0.66 (0.57 - 0.76) | <.001 | |
| **Men** | 131/2955 | 5 (4 - 6) | 43/1037 | 4 (3 - 6) | 0.88 (0.60 - 1.30) | 0.53 | |
| **25-49 years** | 4560/11079 | 41 (40 - 42) | 2085/4932 | 40 (38 – 42) | 0.97 (0.91 - 1.04) | 0.41 | |
| **Women** | 3138/6953 | 45 (44 – 47) | 1421/2949 | 47 (44 - 49) | 1.03 (0.98 - 1.10) | 0.26 | |
| **Men** | 1422/4126 | 35 (34 - 37) | 664/1983 | 31 (28 - 33) | 0.86 (0.78 - 0.95) | 0.003 | |
| **HIV incidence^*^** | | | | | | | |
| **18-24 years** | 62/6949 | 2.9 (2.1 - 3.7) | 9/2333 | 1.0 (0.3 - 1.7) | 0.35 (0.16 - 0.74) | 0.006 | |
| **Women** | 46/3994 | 4.3 (3.0 - 5.7) | 7/1300 | 1.7 (0.4 - 3.0) | 0.40 (0.17 - 0.89) | 0.03 | |
| **Men** | 16/2955 | 1.6 (0.9 - 2.4) | 2/1033 | 0.4 (0.0 - 1.0) | 0.23 (0.04 - 1.30) | 0.10 | |
| **25-49 years** | 52/11026 | 2.1 (1.5 - 2.8) | 16/4931 | 1.5 (0.7 - 2.3) | 0.70 (0.39 - 1.28) | 0.25 | |
| **Women** | 33/6920 | 2.3 (1.5 - 3.1) | 11/2951 | 1.8 (0.6 - 3.0) | 0.79 (0.37 - 1.67) | 0.53 | |
| **Men** | 19/4106 | 2.0 (1.1 - 2.9) | 5/1980 | 1.2 (0.1 - 2.3) | 0.61 (0.22 - 1.70) | 0.35 | |
| **HIV viral load suppression prevalence among all HIV-seropositive adults**^†^ | | | | | |  | |
| **18-24 years** | 214/1160 | 18 (16 - 20) | 142/276 | 50 (44 - 56) | 2.82 (2.36 - 3.36) | <.001 | |
| **Women** | 203/1029 | 19 (17 – 22) | 128/233 | 54 (48 – 61) | 2.83 (2.36 - 3.39) | <.001 | |
| **Men** | 11/131 | 9 (4 – 14) | 14/43 | 32 (17 - 46) | 3.58 (1.73 - 7.40) | 0.0006 | |
| **25-49 years** | 1758/4526 | 39 (37 - 40) | 1567/2085 | 74 (72 - 76) | 1.91 (1.82 - 2.02) | <.001 | |
| **Women** | 1277/3117 | 41 (40 - 43) | 1114/1421 | 78 (76 – 81) | 1.89 (1.79 - 2.00) | <.001 | |
| **Men** | 481/1409 | 35 (32 – 38) | 453/664 | 66 (62 - 70) | 1.90 (1.72 - 2.10) | <.001 | |
| **Unsuppressed HIV viremia prevalence among all, regardless of HIV status**^‡^ | | | | | |  | |
| **18-24 years** | 946/6962 | 13 (12 - 14) | 134/2333 | 5 (4 - 7) | 0.41 (0.34 - 0.50) | <.001 | |
| **Women** | 826/4007 | 21 (20 – 23) | 105/1296 | 8 (6 - 10) | 0.38 (0.30 - 0.47) | <.001 | |
| **Men** | 120/2955 | 4 (3 - 5) | 29/1037 | 3 (2 - 4) | 0.66 (0.42 - 1.04) | 0.07 | |
| **25-49 years** | 2768/11079 | 25 (24 – 26) | 518/4932 | 10 (9 – 12) | 0.42 (0.37 - 0.47) | <.001 | |
| **Women** | 1840/6953 | 26 (25 – 28) | 307/2949 | 10 (9 – 12) | 0.39 (0.33 - 0.45) | <.001 | |
| **Men** | 928/4126 | 23 (21 - 25) | 211/1983 | 11 (9 - 12) | 0.46 (0.39 - 0.54) | <.001 | |

^*^ Measured using LAg avidity assay and HIV viral load

† Defined as proportion of persons with HIV viral load <1000 copies/mL in the population testing HIV seropositive

‡ Defined as proportion of persons with HIV viral load ≥1000 copies/mL in the total population, regardless of HIV status

**S3 Table: HIV care continuum (conditional proportions) by age and gender in 2011 and 2016*.**

|  | **2011** | | **2016** | | **2011 versus 2016** | |
| --- | --- | --- | --- | --- | --- | --- |
|  | **N** | **% (95% CI)** | **N** | **% (95% CI)** | **Risk ratio (95% CI)** | **p-value** |
| **Diagnosed** |  |  |  |  |  |  |
| **Total** | 5,716 | 62 (60, 63) | 2,352 | 84 (82, 86) | 1.37 (1.32, 1.41) | < 0.001 |
| **Women** | 4,166 | 68 (66, 69) | 1,652 | 88 (87, 90) | 1.30 (1.26, 1.34) | < 0.001 |
| **Men** | 1,550 | 50 (47, 52) | 700 | 75 (71, 78) | 1.51 (1.41, 1.62) | < 0.001 |
| **18-24 year olds** | 1,168 | 49 (46, 53) | 275 | 66 (60, 71) | 1.33 (1.19, 1.48) | < 0.001 |
| **25-49 year olds** | 4,548 | 64 (63, 66) | 2,077 | 86 (85, 88) | 1.34 (1.30, 1.39) | < 0.001 |
| **On Treatment** |  |  |  |  |  |  |
| **Total** | 3,571 | 53 (51, 55) | 2,015 | 86 (84, 88) | 1.64 (1.56, 1.71) | < 0.001 |
| **Women** | 2,816 | 49 (47, 51) | 1,471 | 86 (84, 88) | 1.76 (1.68, 1.85) | < 0.001 |
| **Men** | 755 | 63 (59, 67) | 544 | 86 (83, 90) | 1.37 (1.28, 1.48) | < 0.001 |
| **18-24 year olds** | 580 | 31 (27, 35) | 188 | 80 (74, 87) | 2.58 (2.21, 3.00) | < 0.001 |
| **25-49 year olds** | 2,991 | 56 (54, 58) | 1,827 | 87 (85, 89) | 1.54 (1.47, 1.61) | < 0.001 |
| **Virally Suppressed** |  |  |  |  |  |  |
| **Total** | 1,833 | 85 (83, 87) | 1,740 | 91 (90, 93) | 1.08 (1.05, 1.10) | < 0.001 |
| **Women** | 1,364 | 83 (81, 86) | 1,269 | 92 (91, 94) | 1.10 (1.07, 1.14) | < 0.001 |
| **Men** | 469 | 87 (84, 91) | 471 | 89 (86, 92) | 1.02 (0.97, 1.07) | 0.40 |
| **18-24 year olds** | 180 | 64 (57, 71) | 154 | 77 (71, 84) | 1.21 (1.05, 1.39) | 0.01 |
| **25-49 year olds** | 1,653 | 87 (85, 89) | 1,586 | 93 (91, 94) | 1.07 (0.98, 1.16) | 0.10 |
| * Data are presented in bar graph format in Figure 1 of main manuscript | | | | | | |

U**References**

1. Pilcher CD, Fiscus SA, Nguyen TQ, Foust E, Wolf L, Williams D, et al. Detection of acute infections during HIV testing in North Carolina. New England Journal of Medicine. 2005;352(18):1873-83. Epub 2005/05/06. doi: 10.1056/NEJMoa042291. PubMed PMID: 15872202.

2. Pilcher CD, McPherson JT, Leone PA, Smurzynski M, Owen-O'Dowd J, Peace-Brewer AL, et al. Real-time, universal screening for acute HIV infection in a routine HIV counseling and testing population. Journal of the American Medical Association. 2002;288(2):216-21. Epub 2002/07/04. PubMed PMID: 12095386.

3. Duong YT, Kassanjee R, Welte A, Morgan M, De A, Dobbs T, et al. Recalibration of the limiting antigen avidity EIA to determine mean duration of recent infection in divergent HIV-1 subtypes. PloS One. 2015;10(2):e0114947. Epub 2015/02/25. doi: 10.1371/journal.pone.0114947. PubMed PMID: 25710171; PubMed Central PMCID: PMCPMC4339840.

4. Duong YT, Qiu M, De AK, Jackson K, Dobbs T, Kim AA, et al. Detection of recent HIV-1 infection using a new limiting-antigen avidity assay: potential for HIV-1 incidence estimates and avidity maturation studies. PloS One. 2012;7(3):e33328. Epub 2012/04/06. doi: 10.1371/journal.pone.0033328. PubMed PMID: 22479384; PubMed Central PMCID: PMCPMC3314002.

5. Tibshirani R. Regression shrinkage and selection via the Lasso. Journal of the Royal Statistical Society Series B (Methodological). 1996;58(1):267-88.

6. Magidson J. SI-CHAID User's Guide. Belmont, Massachusetts: Statistical Innovations Inc.; 2005.

7. 2017 Population and Housing Census Preliminary Report. Mbabane, Swaziland: Central Statistics Office, 2017.

8. Swaziland Population Projections, 2007-2030: changing shape of the population pyramid. Mbabane, Eswatini: Central Statistical Office, 2010.
